# Supplementary material for: Evaluation of TFR-1 Expression in Feline Mammary Cancer and In Vitro Antitumor Efficacy Study of Doxorubicin-Loaded H-Ferritin Nanocages
Source: Cancers (Basel). 2021 Mar 12;13(6):1248. doi: 10.3390/cancers13061248 (PMC8000254; doi:10.3390/cancers13061248)
Supplement: Supplementary file 1 [file cancers-13-01248-s001.zip › Figure S1.pdf]

invitrogen
by Thermo Fisher Scientific

PRODUCT INFORMATION

iBright™ Prestained Protein Ladder

Pub. No. MAN0016682
Rev. Date 20 July 2017 (Rev. E.00)

XXXXXX

Lot: XXXXXXXX Expiry Date: YYYY-MM-DD

Components	#LC5605	#LC5615
iBright Prestained Protein Ladder	25 µL	2 × 250 µL

Storage Buffer: 62.5 mM Tris•H_3PO_4 (pH 7.5 at 25°C), 1 mM EDTA, 2% (w/v) SDS, 10 mM DTT, 1 mM NaN_3 and 33% (v/v) glycerol.

Storage: Upon receipt store at -20°C. Product is shipped on dry ice.

thermofisher.com

For Research Use Only. Not for use in diagnostic procedures.

Description

Invitrogen™ iBright™ Prestained Protein Ladder is a mixture of 12 proteins ranging from 11 kDa to 250 kDa (11, 15, 26, 30, 34, 43, 55, 70, 80, 95, 130 and 250 kDa). The protein ladder produces 10 well-defined blue pre-stained bands by SDS-PAGE and 2 unstained bands (30 kDa and 80 kDa) containing IgG binding sites, which can be used for confirmation of Western blot success (see website for product images). The prestained bands of the iBright protein ladder can be visualized by SDS-PAGE or fluorescently viewed using a fluorescent detection instrument (e.g., Amersham™ Typhoon™ or LI-COR™ Odyssey™ imagers). For an easy reference, the 55 kDa protein band has a greater intensity than the other prestained proteins in the ladder.

The 30 kDa and 80 kDa immunodetectable bands contain IgG binding sites that can be visualized simultaneously with your target protein using the same antibody conjugate and protocol (see website for product images). These two immunodetectable bands are compatible with chemiluminescent or fluorescent detection methods for Western blot analysis. The iBright protein ladder can be visualized on gels using Thermo Scientific™ PageBlue™, SimplyBlue™ SafeStain, NBS Biologicals™ SafeBlue™, or other Coomassie stains, or directly on the membrane using Novex™ Reversible Membrane or Pierce™ Reversible protein stains. The iBright protein ladder provides convenient visualization during electrophoresis and transfer, and clear, sharp confirmation of Western blot detection of your target protein.

The protein ladder is conveniently packaged and ready to use with no heating, diluting, or additional reducing agent required.

Rev. E.00 V

Important Product Information

- Do not boil the protein ladder.
- Use a Coomassie stain (e.g., PageBlue stain, SimplyBlue SafeStain, etc.) or reversible membrane stain (e.g., Pierce Reversible protein stain) to view the 30 and 80 kDa bands with the visible bands.
- In low-percentage gels (< 10%), the low molecular weight proteins in the ladder may migrate with the dye front.
- Large proteins (> 100 kDa) in the ladder may require longer transfer times or higher transfer voltages for Western blotting.
- The mobility of prestained proteins can vary in different SDS-PAGE buffer systems; however, they are suitable for approximate molecular weight determination when calibrated against unstained standards in the same system.
- An additional low molecular weight band of free dye is visible when using fluorescent detection.
- The apparent molecular weight variance of the 10 prestained proteins is ~5%.
- Intensity of 30 kDa and 80 kDa immunodetectable bands depends on secondary antibody concentration and substrate sensitivity.
- Primary antibodies with low starting concentrations may result in insufficient chemiluminescent detection of the Western blot positive control bands. If unstained 30 kDa and 80 kDa bands produce weak or no signal, spike the diluted primary antibody with the corresponding Rabbit IgG or Mouse IgG to a concentration of 1-5 µg/mL, prior to secondary antibody incubation. Follow with respective secondary (GAM/GAR) incubation to increase the intensity of Western blot positive control bands in the iBright Prestained Protein Ladder.

Procedure for using iBright protein ladder in polyacrylamide gel electrophoresis

1. Thaw the ladder at room temperature.
2. Vortex gently to ensure the solution is homogeneous.
3. Load the ladder on the gel (see Table 1 for recommended volumes).
4. Return unused protein ladder to -20°C and store up to one year.
5. Visualize the ladder via fluorescent detection using a fluorescent imager (e.g., Typhoon or LI-COR Odyssey imagers).
6. Detect the 30 kDa and 80 kDa ladder bands on membranes using the same alkaline phosphatase or horseradish peroxidase-conjugated antibody with chemiluminescent substrates, or fluorescently-labeled antibodies as used for your target protein.

Table 1. Volumes of Invitrogen iBright Prestained Protein Ladder to load for different applications.

Gel type	Visual detection (1.0 mm gel thickness)	Fluorescent detection	Chemiluminescent detection
Mini gel	1-3 µL (12-well & 20-well)<br>2 µL (26-well)	1-3 µL (12-well & 20-well)<br>2 µl (26-well)	1-3 µL (12-well & 20-well)<br>2 µL (26-well)
Midi gel	2-4 µL (12-well & 20-well)<br>2 µL (26-well)	2-3 µL (12-well & 20-well)<br>2 µL (26-well)	2-3 µL (12-well & 20-well)<br>2 µL (26-well)

Table 2. Example migration patterns for various gel types.

Migration Patterns of iBright™ Prestained Protein Ladder	Invitrogen™ iBright™ Prestained Protein Ladder											
**Gel type**	**Tris-Glycine†**				**Tris-Acetate††**		**Bis-Tris††**					
**Gel concentration**	4-20%	4-12%	10%	12%	3-8%	7%	4-12%		10%		12%	
**Running buffer**	Tris-Glycine				Tris-Acetate		MOPS*	MES	MOPS	MES	MOPS	MES
	**Apparent Molecular Sizes (kDa)**											
**% length of gel**<br>10<br>20<br>30<br>40<br>50<br>60<br>70<br>80<br>90<br>100	250<br>130<br>90<br>80<br>70<br>55<br>43<br>34<br>30<br>26<br>15<br>10	250<br>130<br>90<br>80<br>70<br>55<br>43<br>34<br>30<br>26<br>15<br>10	250<br>130<br>90<br>80<br>70<br>55<br>43<br>34<br>30<br>26<br>15	250<br>130<br>90<br>80<br>70<br>55<br>43<br>34<br>30<br>26<br>15<br>10	205<br>120<br>85<br>80<br>62<br>50<br>40<br>33<br>30	205<br>120<br>85<br>80<br>62<br>50<br>40<br>33	195<br>119<br>84<br>80<br>57<br>50<br>39<br>28<br>27<br>22<br>16<br>10	199<br>119<br>84<br>83<br>57<br>50<br>39<br>28<br>27<br>22<br>15<br>10	199<br>119<br>84<br>83<br>57<br>50<br>39<br>28<br>27<br>22<br>15<br>10	120<br>85<br>84<br>60<br>50<br>40<br>28<br>27<br>24<br>15<br>11	120<br>85<br>84<br>60<br>50<br>40<br>28<br>27<br>24<br>15<br>11	119<br>84<br>83<br>59<br>50<br>39<br>28<br>23<br>15<br>14<br>10

† Migration patterns were determined using respective Novex™ Wedgewell

†† Migration patterns were determined using respective NuPAGE™ precast gels.

* Migration pattern for 4-12% Bis-Tris in MOPS buffer measured using 4-12% Bolt™ Bis-Tris precast gel.

Related Thermo Scientific Products

34580	SuperSignal™ West Pico PLUS Chemiluminescent Substrate, 500 mL
34075	SuperSignal™ West Dura Chemiluminescent Substrate, 100 mL
34096	SuperSignal™ West Femto Chemiluminescent Substrate, 200 mL
32430	Stabilized Goat Anti-Mouse IgG (H+L), Peroxidase Conjugated (10 µg/mL), 2 mL
32460	Stabilized Goat Anti-Rabbit IgG (H+L), Peroxidase Conjugated (10 µg/mL), 2 mL
46430	Restore™ PLUS Western Blot Stripping Buffer, 500 mL
46640	SuperSignal™ Western Blot Enhancer
37542	StartingBlock™ (TBS) Blocking Buffer
37578	StartingBlock™ (PBS) Blocking Buffer

LIMITED USE LABEL LICENSE: Internal Research and Development Use Only.

The purchase of this product conveys to the buyer the limited, non-exclusive, non-transferable right (without the right to resell, repackage, or further sublicense) to use this product for internal research and development purposes. No other license is granted to the buyer whether expressly, by implication, by estoppel or otherwise. In particular, the purchase of the product does not include or carry any right or license to use, develop, or otherwise exploit this product commercially and no rights are conveyed to the buyer to use the product or components of the product for purposes including but not limited to provision of services to a third party, generation of commercial databases or clinical diagnostics. This product is sold pursuant to authorization from Thermo Fisher Scientific and Thermo Fisher Scientific reserves all other rights. For information on purchasing a license for uses other than internal research and development purposes, please contact outlicensing@lifetech.com or Out Licensing, Life Technologies Inc., 5781 Van Allen Way, Carlsbad, California 92008.

PRODUCT USE LIMITATION

This product is developed, designed and sold exclusively *for research purposes and in vitro use only.* The product was not tested for use in diagnostics or for drug development, nor is it suitable for administration to humans or animals.
Please refer to thermofisher.com for Material Safety Data Sheet of the product.

Manufacturing site. Thermo Fisher Scientific Baltics UAB. V.A Graiciuno 8, LT-02241. Vilnius, Lithuania.

© 2017 Thermo Fisher Scientific Inc. All rights reserved. Typhoon is a trademark of General Electric. Odyssey is a trademark of LI-COR Inc. All other trademarks are the property of Thermo Fisher Scientific Inc. and its subsidiaries.
