# Supplementary material for: Evaluation of TFR-1 Expression in Feline Mammary Cancer and In Vitro Antitumor Efficacy Study of Doxorubicin-Loaded H-Ferritin Nanocages
Source: Cancers (Basel). 2021 Mar 12;13(6):1248. doi: 10.3390/cancers13061248 (PMC8000254; doi:10.3390/cancers13061248)
Supplement: Supplementary file 1 [file cancers-13-01248-s001.zip › Figure S4.pdf]

Website: thermofisher.com
Customer Service (US): 1 800 955 6288 ext. 1
Technical Support (US): 1 800 955 6288 ext. 441
thermofisher.com/contactus

Performance guaranteed

Transferrin Receptor Monoclonal Antibody (H68.4)

Catalog Number 13-6890

Product data sheet

Details

**Size**	500 µg
**Host/Isotope**	Mouse / IgG1
**Class**	Monoclonal
**Type**	Antibody
**Clone**	H68.4
**Immunogen**	Recombinant human transferrin receptor.
**Conjugate**	Unconjugated
**Form**	Liquid
**Concentration**	0.5 mg/mL
**Purification**	Affinity chromatography
**Storage buffer**	PBS, pH 7.4
**Contains**	0.1% sodium azide
**Storage Conditions**	-20°C

Species Reactivity

**Species reactivity**	Chicken, Hamster, Human, Mouse, Rat
**Published species**	Dog, Yeast, Rat, Pig, Non-human primate, Hamster, Cat, Human, Mouse, Chicken, Not Applicable, Horse

Tested Applications	Dilution *
**ELISA (ELISA)**	1-5 µg/mL
**Immunocytochemistry (ICC)**	1:250
**Immunofluorescence (IF)**	1:250
**Immunohistochemistry (IHC)**	Assay Dependent
**Immunomicroscopy (IM)**	Assay Dependent
**Immunoprecipitation (IP)**	2-5 µg
**Western Blot (WB)**	1 µg/mL

Published Applications

**Western Blot (WB)**	See 62 publications below
**Immunocytochemistry (ICC)**	See 28 publications below
**Immunohistochemistry (Paraffin) (IHC (P))**	See 3 publications below
**Miscellaneous PubMed (MISC)**	See 70 publications below
**Flow Cytometry (Flow)**	See 5 publications below
**Immunoprecipitation (IP)**	See 1 publications below

* Suggested working dilutions are given as a guide only. It is recommended that the user titrate the product for use in their own experiment using appropriate negative and positive controls.

Product specific information

Mouse anti-CD71 (H68.4) antibody reacts with the N-terminal region of the human transferrin receptor. Due to the conserved amino acid sequences of the cytoplasmic tail among species, this antibody is able to cross-react with chicken, mouse and Chinese hamster CD71. Cross-reactivity has also been observed with rat adrenal pheochromocytoma (PC-12).

Background/Target Information

The human transferrin receptor (TR; CD71) is a homodimeric type II transmembrane glycoprotein consisting of two identical 95 kDa subunits covalently linked by intermolecular disulfide bonds in erythroid blood cell line and in activated leukocytes. CD71 has an extracellular domain of 671 amino acids, a single 28-residue transmembrane region, and a 61-residue N-terminal cytoplasmic domain. Expression of the receptor is limited on normal tissue, however, highly expressed on the surface of some tumors. CD71 is included in the class of ligand transport receptors which are internalized by clathrin-mediated endocytosis. An important function of the receptor is to bind the serum transport protein transferrin (TF; apotransferrin) and mediate the uptake of iron into the cell. Acidification of endosomes by vesicular membrane proton pumps leads to dissociation of iron ions, whereas transferrin (apotransferrin) remains associated with CD71 and recycles to the cell surface, where it is released upon exposure to normal pH. CD71 is also involved in uptake of non-transferrin bound iron. A growing area of research involves the use of monoclonal anti-CD71 antibodies to inhibit the uptake of iron and thus block tumor cell growth.

For Research Use Only. Not for use in diagnostic procedures. Not for resale without express authorization.

Products are warranted to operate or perform substantially in conformance with published Product specifications in effect at the time of sale, as set forth in the Production documentation, specifications and/or accompanying package inserts ("Documentation"). No claim of suitability for use in applications regulated by FDA is made. The warranty provided herein is valid only when used by properly trained individuals. Unless otherwise stated in the Documentation, this warranty is limited to one year from date of shipment when the Product is subjected to normal, proper and intended usage. This warranty does not extend to anyone other than the Buyer. Any model or sample furnished to Buyer is merely illustrative of the general type and quality of goods and does not represent that any Product will conform to such model or sample.

NO OTHER WARRANTIES, EXPRESS OR IMPLED, ARE GRANTED INCLUDING WITHOUT LIMITATION, IMPLIED WARRANTIES OF MERCHANTABILITY, FITNESS FOR ANY PARTICULAR PURPOSE, OR NON INFRINGEMENT. BUYER'S EXCLUSIVE REMEDY FOR NON-CONFORMING PRODUCTS DURING THE WARRANTY PERIOD IS LIMITED TO REPAIR, REPLACEMENT OF OR REFUND FOR THE NON-CONFORMING PRODUCT(S) AT SELLER'S SOLE OPTION. THERE IS NO OBLIGATION TO REPAIR, REPLACE OR REFUND FOR PRODUCTS AS THE RESULT OF (I) ACCIDENT, DISASTER OR EVENT OF FORCE MAJEURE, (II) MISUSE, FAULT OR NEGLIGENCE OF OR BY BUYER, (III) USE OF THE PRODUCTS IN A MANNER FOR WHICH THEY WERE NOT DESIGNED, OR (IV) IMPROPER STORAGE AND HANDLING OF THE PRODUCTS. Unless otherwise expressly stated on the Product or in the documentation accompanying the Product, the Product is intended for research only and is not to be used for any other purpose, including without limitation, unauthorized commercial uses, in vitro diagnostic uses, ex vivo or in vivo therapeutic uses, or any type of consumption by or application to human or animals.

For Research Use Only. Not for use in diagnostic procedures. Not for resale without express authorization.

Products are warranted to operate or perform substantially in conformance with published Product specifications in effect at the time of sale, as set forth in the Production documentation, specifications and/or accompanying package inserts ("Documentation"). No claim of suitability for use in applications regulated by FDA is made. The warranty provided herein is valid only when used by properly trained individuals. Unless otherwise stated in the Documentation, this warranty is limited to one year from date of shipment when the Product is subjected to normal, proper and intended usage. This warranty does not extend to anyone other than the Buyer. Any model or sample furnished to Buyer is merely illustrative of the general type and quality of goods and does not represent that any Product will conform to such model or sample.

NO OTHER WARRANTIES, EXPRESS OR IMPLED, ARE GRANTED INCLUDING WITHOUT LIMITATION, IMPLIED WARRANTIES OF MERCHANTABILITY, FITNESS FOR ANY PARTICULAR PURPOSE, OR NON INFRINGEMENT. BUYER'S EXCLUSIVE REMEDY FOR NON-CONFORMING PRODUCTS DURING THE WARRANTY PERIOD IS LIMITED TO REPAIR, REPLACEMENT OF OR REFUND FOR THE NON-CONFORMING PRODUCT(S) AT SELLER'S SOLE OPTION. THERE IS NO OBLIGATION TO REPAIR, REPLACE OR REFUND FOR PRODUCTS AS THE RESULT OF (I) ACCIDENT, DISASTER OR EVENT OF FORCE MAJEURE, (II) MISUSE, FAULT OR NEGLIGENCE OF OR BY BUYER, (III) USE OF THE PRODUCTS IN A MANNER FOR WHICH THEY WERE NOT DESIGNED, OR (IV) IMPROPER STORAGE AND HANDLING OF THE PRODUCTS. Unless otherwise expressly stated on the Product or in the documentation accompanying the Product, the Product is intended for research only and is not to be used for any other purpose, including without limitation, unauthorized commercial uses, in vitro diagnostic uses, ex vivo or in vivo therapeutic uses, or any type of consumption by or application to human or animals.

Product Images For Transferrin Receptor Monoclonal Antibody (H68.4)

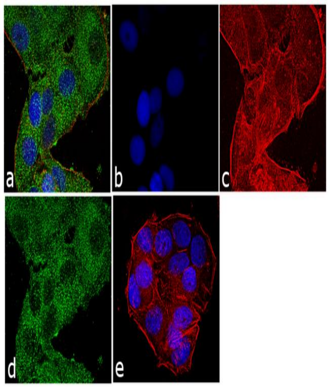

Transferrin Receptor Antibody (13-6890) in IF

Immunofluorescence analysis of Transferrin Receptor Monoclonal Antibody (H68.4) was performed using70% confluent log phase MCF-7 cells. The cells were fixed with 4% paraformaldehyde for 10 minutes, permeabilized with 0.1% Triton™ X-100 for 10 minutes, and blocked with 1% BSA for 1 hour at room temperature. The cells were labeled with Transferrin Receptor (H68.4) Mouse Monoclonal Antibody (Product # 13-6890) at 1:250 dilution in 0.1% BSA and incubated for 3 hours at room temperature and then labeled with Goat anti-Mouse IgG (H+L) Superclonal™ Secondary Antibody, Alexa Fluor® 488 conjugate (Product # A28175) at a dilution of 1:2000 for 45 minutes at room temperature (Panel a: green). Nuclei (Panel b: blue) were stained with SlowFade® Gold Antifade Mountant with DAPI (Product # S36938). F-actin (Panel c: red) was stained with Rhodamine Phalloidin (Product # R415, 1:300). Panel d represents the merged image showing cytoplasmic localization. Panel e shows the no primary antibody control. The images were captured at 60X magnification.

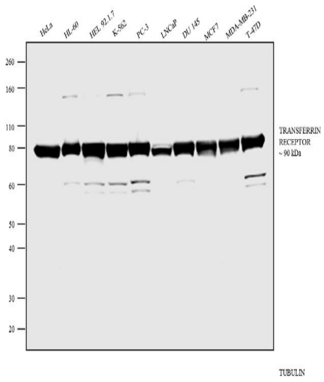

Transferrin Receptor Antibody (13-6890) in WB

Western blot analysis was performed on whole cell extracts (30 µg lysate) of HeLa (Lane 1), HL-60 (Lane 2), HEL 92.1.7 (Lane 3), K-562 (Lane 4), PC-3 (Lane 5), LNCaP (Lane 6), DU 145 (Lane 7), MCF7 (Lane 8), MDA-MB-231 (Lane 9) and T-47D (Lane 10). The blot was probed with Anti-Transferrin Receptor Mouse Monoclonal Antibody (Product # 13-6890, 2 µg /mL) and detected by chemiluminescence using Goat anti-Mouse IgG (H+L) Superclonal™ Secondary Antibody, HRP conjugate (Product # A28177, 0.4 µg/mL, 1:2500 dilution). A 90 kDa band corresponding to Transferrin Receptor was observed across the cell lines tested. Known quantity of protein samples were electrophoresed using Novex®NuPAGE®4-12 % Bis-Tris gel (Product # NP0321BOX), XCell SureLock™ Electrophoresis System (Product # EI0002) and Novex® Sharp Pre-Stained Protein Standard (Product # LC5800). Resolved proteins were then transferred onto a nitrocellulose membrane with iBlot® 2 Dry Blotting System (Product # IB21001). The membrane was probed with the relevant primary and secondary Antibody following blocking with 5 % skimmed milk. Chemiluminescent detection was performed using Novex® ECL Chemiluminescent Substrate Reagent Kit (Product # WP20005).

For Research Use Only. Not for use in diagnostic procedures. Not for resale without express authorization.

Products are warranted to operate or perform substantially in conformance with published Product specifications in effect at the time of sale, as set forth in the Production documentation, specifications and/or accompanying package inserts ("Documentation"). No claim of suitability for use in applications regulated by FDA is made. The warranty provided herein is valid only when used by properly trained individuals. Unless otherwise stated in the Documentation, this warranty is limited to one year from date of shipment when the Product is subjected to normal, proper and intended usage. This warranty does not extend to anyone other than the Buyer. Any model or sample furnished to Buyer is merely illustrative of the general type and quality of goods and does not represent that any Product will conform to such model or sample.

NO OTHER WARRANTIES, EXPRESS OR IMPLED, ARE GRANTED INCLUDING WITHOUT LIMITATION, IMPLIED WARRANTIES OF MERCHANTABILITY, FITNESS FOR ANY PARTICULAR PURPOSE, OR NON INFRINGEMENT. BUYER'S EXCLUSIVE REMEDY FOR NON-CONFORMING PRODUCTS DURING THE WARRANTY PERIOD IS LIMITED TO REPAIR, REPLACEMENT OF OR REFUND FOR THE NON-CONFORMING PRODUCT(S) AT SELLER'S SOLE OPTION. THERE IS NO OBLIGATION TO REPAIR, REPLACE OR REFUND FOR PRODUCTS AS THE RESULT OF (I) ACCIDENT, DISASTER OR EVENT OF FORCE MAJEURE, (II) MISUSE, FAULT OR NEGLIGENCE OF OR BY BUYER, (III) USE OF THE PRODUCTS IN A MANNER FOR WHICH THEY WERE NOT DESIGNED, OR (IV) IMPROPER STORAGE AND HANDLING OF THE PRODUCTS. Unless otherwise expressly stated on the Product or in the documentation accompanying the Product, the Product is intended for research only and is not to be used for any other purpose, including without limitation, unauthorized commercial uses, in vitro diagnostic uses, ex vivo or in vivo therapeutic uses, or any type of consumption by or application to human or animals.

PubMed References For Transferrin Receptor Monoclonal Antibody (H68.4)

62 Western Blot References

Species / Dilution	Summary
Not Applicable / Not Cited	13-6890 was used in western blot to elucidate the compartmentalization of breast cancer resistance protein ABCG2 in the plasma membrane and the influence of membrane cholesterol on its efflux activity<br><br>The Journal of pharmacology and experimental therapeutics (Oct 2007; 323: 257)<br>**"Localization of the human breast cancer resistance protein (BCRP/ABCG2) in lipid rafts/caveolae and modulation of its activity by cholesterol in vitro."**<br>Author(s):Storch CH,Ehehalt R,Haefeli WE,Weiss J<br>PubMed Article URL:http://dx.doi.org/10.1124/jpet.107.122994
Mouse / Not Cited	13-6890 was used in western blot to investigate the convergence of APP and BACE-.<br><br>Neuron (Aug 2013; 79: 447)<br>**"Activity-induced convergence of APP and BACE-1 in acidic microdomains via an endocytosis-dependent pathway."**<br>Author(s):Das U,Scott DA,Ganguly A,Koo EH,Tang Y,Roy S<br>PubMed Article URL:http://dx.doi.org/10.1016/j.neuron.2013.05.035
Rat / Not Cited	13-6890 was used in western blot to investigate the function of protein kinase CK2 in lipid rafts.<br><br>FEBS letters (Jan 2011; 585: 414)<br>**"Protein kinase CK2 associates to lipid rafts and its pharmacological inhibition enhances neurotransmitter release."**<br>Author(s):Gil C,Falqués A,Sarró E,Cubi R,Blasi J,Aguilera J,Itarte E<br>PubMed Article URL:http://dx.doi.org/10.1016/j.febslet.2010.12.029
Not Applicable / 1:500	13-6890 was used in western blot to test if the extracellular matrix metalloproteinase inducer takes part in the induction of proteolytic enzymes in the rat tooth germ<br><br>Histochemistry and cell biology (Sep 2007; 128: 195)<br>**"Immunocytochemical and biochemical detection of EMMPRIN in the rat tooth germ: differentiation-dependent co-expression with MMPs and co-localization with caveolin-1 in membrane rafts of dental epithelial cells."**<br>Author(s):Schwab W,Harada H,Goetz W,Nowicki M,Witt M,Kasper M,Barth K<br>PubMed Article URL:http://dx.doi.org/10.1007/s00418-007-0313-7
Not Applicable / Not Cited	13-6890 was used in western blot to elucidate how endoplasmic reticulum-to-Golgi transport is blocked during mitosis<br><br>FEBS letters (Mar 2004; 561: 44)<br>**"Regulation of a COPII component by cytosolic O-glycosylation during mitosis."**<br>Author(s):Dudognon P,Maeder-Garavaglia C,Carpentier JL,Paccaud JP<br>PubMed Article URL:http://dx.doi.org/10.1016/S0014-5793(04)00109-7
Not Applicable / Not Cited	13-6890 was used in western blot to identify which guanine nucleotide exchange factors mediate the functional interaction of HIV-1 Nef with p21-activate kinase 2 activity<br><br>Journal of virology (Mar 2008; 82: 2918)<br>**"Human immunodeficiency virus type 1 Nef recruits the guanine exchange factor Vav1 via an unexpected interface into plasma membrane microdomains for association with p21-activated kinase 2 activity."**<br>Author(s):Rauch S,Pulkkinen K,Saksela K,Fackler OT<br>PubMed Article URL:http://dx.doi.org/10.1128/JVI.02185-07
Not Applicable / Not Cited	13-6890 was used in western blot to use free-flow electrophoresis for the charge-based separation of detergent-resistant membranes<br><br>Biochemical and biophysical research communications (Jul 2004; 319: 826)<br>**"Charge-based separation of detergent-resistant membranes of mouse splenic B cells."**<br>Author(s):Katsumata O,Kimura T,Nagatsuka Y,Hirabayashi Y,Sugiya H,Furuyama S,Yanagishita M,Hara-Yokoyama M<br>PubMed Article URL:http://dx.doi.org/10.1016/j.bbrc.2004.05.058
Not Applicable / Not Cited	13-6890 was used in western blot to examine AP-3-dependent synaptic vesicle biogenesis<br><br>The Journal of neuroscience : the official journal of the Society for Neuroscience (Jan 2010; 30: 820)<br>**"Hermansky-Pudlak protein complexes, AP-3 and BLOC-1, differentially regulate presynaptic composition in the striatum and hippocampus."**<br>Author(s):Newell-Litwa K,Chintala S,Jenkins S,Pare JF,McGaha L,Smith Y,Faundez V<br>PubMed Article URL:http://dx.doi.org/10.1523/JNEUROSCI.3400-09.2010

For Research Use Only. Not for use in diagnostic procedures. Not for resale without express authorization.

Products are warranted to operate or perform substantially in conformance with published Product specifications in effect at the time of sale, as set forth in the Production documentation, specifications and/or accompanying package inserts ("Documentation"). No claim of suitability for use in applications regulated by FDA is made. The warranty provided herein is valid only when used by properly trained individuals. Unless otherwise stated in the Documentation, this warranty is limited to one year from date of shipment when the Product is subjected to normal, proper and intended usage. This warranty does not extend to anyone other than the Buyer. Any model or sample furnished to Buyer is merely illustrative of the general type and quality of goods and does not represent that any Product will conform to such model or sample.

NO OTHER WARRANTIES, EXPRESS OR IMPLED, ARE GRANTED INCLUDING WITHOUT LIMITATION, IMPLIED WARRANTIES OF MERCHANTABILITY, FITNESS FOR ANY PARTICULAR PURPOSE, OR NON INFRINGEMENT. BUYER'S EXCLUSIVE REMEDY FOR NON-CONFORMING PRODUCTS DURING THE WARRANTY PERIOD IS LIMITED TO REPAIR, REPLACEMENT OF OR REFUND FOR THE NON-CONFORMING PRODUCT(S) AT SELLER'S SOLE OPTION. THERE IS NO OBLIGATION TO REPAIR, REPLACE OR REFUND FOR PRODUCTS AS THE RESULT OF (I) ACCIDENT, DISASTER OR EVENT OF FORCE MAJEURE, (II) MISUSE, FAULT OR NEGLIGENCE OF OR BY BUYER, (III) USE OF THE PRODUCTS IN A MANNER FOR WHICH THEY WERE NOT DESIGNED, OR (IV) IMPROPER STORAGE AND HANDLING OF THE PRODUCTS. Unless otherwise expressly stated on the Product or in the documentation accompanying the Product, the Product is intended for research only and is not to be used for any other purpose, including without limitation, unauthorized commercial uses, in vitro diagnostic uses, ex vivo or in vivo therapeutic uses, or any type of consumption by or application to human or animals.

Not Applicable / Not Cited	13-6890 was used in western blot to explore the intracellular transport of occludin in fibroblastic and epithelial cells<br><br>Methods in molecular biology (Clifton, N.J.) (May 2008; 440: 89)<br>**"Cell-surface biotinylation to study endocytosis and recycling of occludin."**<br>Author(s):Nishimura N,Sasaki T<br>PubMed Article URL:http://dx.doi.org/10.1007/978-1-59745-178-9_7
Not Applicable / 1:2000	13-6890 was used in western blot to analyze alteration of fetal iron distribution and elevation of hepatic hepcidin in a rat model of fetal alcohol spectrum disorders caused by prenatal alcohol exposure<br><br>The Journal of nutrition (Jun 2016; 146: 1180)<br>**"Prenatal Alcohol Exposure Alters Fetal Iron Distribution and Elevates Hepatic Hepcidin in a Rat Model of Fetal Alcohol Spectrum Disorders."**<br>Author(s):Huebner SM,Blohowiak SE,Kling PJ,Smith SM<br>PubMed Article URL:http://dx.doi.org/10.3945/jn.115.227983
Not Applicable / Not Cited	13-6890 was used in western blot to elucidate Nef-mediated signaling<br><br>Journal of immunology (Baltimore, Md. : 1950) (Dec 2008; 181: 8425)<br>**"Induction of HIV transcription by Nef involves Lck activation and protein kinase C theta raft recruitment leading to activation of ERK1/2 but not NF kappa B."**<br>Author(s):Witte V,Laffert B,Gintschel P,Krautkrämer E,Blume K,Fackler OT,Baur AS<br>PubMed Article URL:http://dx.doi.org/null
Human / Not Cited	13-6890 was used in western blot to characterize the effects of iron chelators on cell cycle arrest.<br><br>Carcinogenesis (Jun 2003; 24: 1045)<br>**"Potent iron chelators increase the mRNA levels of the universal cyclin-dependent kinase inhibitor p21(CIP1/WAF1), but paradoxically inhibit its translation: a potential mechanism of cell cycle dysregulation."**<br>Author(s):Le NT,Richardson DR<br>PubMed Article URL:http://dx.doi.org/10.1093/carcin/bgg042
Not Applicable / Not Cited	13-6890 was used in western blot to investigate the consequences of Homer proteins interacting with the amyloid precursor protein<br><br>Neurobiology of disease (Jun 2008; 30: 353)<br>**"Homer2 and Homer3 interact with amyloid precursor protein and inhibit Abeta production."**<br>Author(s):Parisiadou L,Bethani I,Michaki V,Krousti K,Rapti G,Efthimiopoulos S<br>PubMed Article URL:http://dx.doi.org/10.1016/j.nbd.2008.02.004
Not Applicable / Not Cited	13-6890 was used in western blot to study the luminal/abluminal localization of a number of blood-brain barrier transporters expressed by endothelial cells<br><br>Neuroscience (Aug 2008; 155: 423)<br>**"Subcellular localization of transporters along the rat blood-brain barrier and blood-cerebral-spinal fluid barrier by in vivo biotinylation."**<br>Author(s):Roberts LM,Black DS,Raman C,Woodford K,Zhou M,Haggerty JE,Yan AT,Cwirla SE,Grindstaff KK<br>PubMed Article URL:http://dx.doi.org/10.1016/j.neuroscience.2008.06.015
Not Applicable / Not Cited	13-6890 was used in western blot to elucidate how nerve growth factor signaling affects chronic myelogenous leukemia cell lines<br><br>Oncogene (Aug 2008; 27: 4678)<br>**"Inhibition of Abl tyrosine kinase enhances nerve growth factor-mediated signaling in Bcr-Abl transformed cells via the alteration of signaling complex and the receptor turnover."**<br>Author(s):Koch A,Scherr M,Breyer B,Mancini A,Kardinal C,Battmer K,Eder M,Tamura T<br>PubMed Article URL:http://dx.doi.org/10.1038/onc.2008.107
Human / Not Cited	136890 was used in immunocytochemistry and western blot to demonstrate that ATP9A has an important role in recycling from endosomes to the plasma membrane<br><br>Molecular biology of the cell (Dec 2016; 27: 3883)<br>**"The phospholipid flippase ATP9A is required for the recycling pathway from the endosomes to the plasma membrane."**<br>Author(s):Tanaka Y,Ono N,Shima T,Tanaka G,Katoh Y,Nakayama K,Takatsu H,Shin HW<br>PubMed Article URL:http://dx.doi.org/10.1091/mbc.E16-08-0586

For Research Use Only. Not for use in diagnostic procedures. Not for resale without express authorization.

Products are warranted to operate or perform substantially in conformance with published Product specifications in effect at the time of sale, as set forth in the Production documentation, specifications and/or accompanying package inserts ("Documentation"). No claim of suitability for use in applications regulated by FDA is made. The warranty provided herein is valid only when used by properly trained individuals. Unless otherwise stated in the Documentation, this warranty is limited to one year from date of shipment when the Product is subjected to normal, proper and intended usage. This warranty does not extend to anyone other than the Buyer. Any model or sample furnished to Buyer is merely illustrative of the general type and quality of goods and does not represent that any Product will conform to such model or sample.

NO OTHER WARRANTIES, EXPRESS OR IMPLED, ARE GRANTED INCLUDING WITHOUT LIMITATION, IMPLIED WARRANTIES OF MERCHANTABILITY, FITNESS FOR ANY PARTICULAR PURPOSE, OR NON INFRINGEMENT. BUYER'S EXCLUSIVE REMEDY FOR NON-CONFORMING PRODUCTS DURING THE WARRANTY PERIOD IS LIMITED TO REPAIR, REPLACEMENT OF OR REFUND FOR THE NON-CONFORMING PRODUCT(S) AT SELLER'S SOLE OPTION. THERE IS NO OBLIGATION TO REPAIR, REPLACE OR REFUND FOR PRODUCTS AS THE RESULT OF (I) ACCIDENT, DISASTER OR EVENT OF FORCE MAJEURE, (II) MISUSE, FAULT OR NEGLIGENCE OF OR BY BUYER, (III) USE OF THE PRODUCTS IN A MANNER FOR WHICH THEY WERE NOT DESIGNED, OR (IV) IMPROPER STORAGE AND HANDLING OF THE PRODUCTS. Unless otherwise expressly stated on the Product or in the documentation accompanying the Product, the Product is intended for research only and is not to be used for any other purpose, including without limitation, unauthorized commercial uses, in vitro diagnostic uses, ex vivo or in vivo therapeutic uses, or any type of consumption by or application to human or animals.

Human / 1:500	13-6890 was used in immunohistochemistry - paraffin section and western blot to test if TfR1/CD71 is differentially expressed among benign and malignant thyroid tissues.<br><br>Thyroid : official journal of the American Thyroid Association (Mar 2011; 21: 267)<br>**"Aberrant expression of TfR1/CD71 in thyroid carcinomas identifies a novel potential diagnostic marker and therapeutic target."**<br>Author(s):Magro G,Cataldo I,Amico P,Torrisi A,Vecchio GM,Parenti R,Asioli S,Recupero D,D'Agata V,Mucignat MT,Perris R<br>PubMed Article URL:http://dx.doi.org/10.1089/thy.2010.0173
Not Applicable / Not Cited	13-6890 was used in western blot to analyze the role of Pael receptor expression in regulating dopamine levels in dopaminergic neurons<br><br>Neuroscience research (Dec 2007; 59: 413)<br>**"Pael receptor is involved in dopamine metabolism in the nigrostriatal system."**<br>Author(s):Imai Y,Inoue H,Kataoka A,Hua-Qin W,Masuda M,Ikeda T,Tsukita K,Soda M,Kodama T,Fuwa T,Honda Y,Kaneko S,Matsumoto S,Wakamatsu K,Ito S,Miura M,Aosaki T,Itohara S,Takahashi R<br>PubMed Article URL:http://dx.doi.org/10.1016/j.neures.2007.08.005
Not Applicable / Not Cited	13-6890 was used in western blot to examine the effect of histone deacetylase 6 reduction on lung cancer cells<br><br>Biochemical and biophysical research communications (Sep 2008; 374: 84)<br>**"Effects of downregulated HDAC6 expression on the proliferation of lung cancer cells."**<br>Author(s):Kamemura K,Ito A,Shimazu T,Matsuyama A,Maeda S,Yao TP,Horinouchi S,Khochbin S,Yoshida M<br>PubMed Article URL:http://dx.doi.org/10.1016/j.bbrc.2008.06.092
Mouse / Not Cited	13-6890 was used in western blot to study the pathogenesis of cardiomyopathy in Friedreich ataxia<br><br>The American journal of pathology (Sep 2013; 183: 745)<br>**"Molecular and functional alterations in a mouse cardiac model of Friedreich ataxia: activation of the integrated stress response, eIF2 phosphorylation, and the induction of downstream targets."**<br>Author(s):Huang ML,Sivagurunathan S,Ting S,Jansson PJ,Austin CJ,Kelly M,Semsarian C,Zhang D,Richardson DR<br>PubMed Article URL:http://dx.doi.org/10.1016/j.ajpath.2013.05.032
Not Applicable / Not Cited	13-6890 was used in western blot to study sonic hedgehog expression in developing and adult hamster brains<br><br>The European journal of neuroscience (Sep 2001; 14: 839)<br>**"High expression and anterograde axonal transport of aminoterminal sonic hedgehog in the adult hamster brain."**<br>Author(s):Traiffort E,Moya KL,Faure H,Hässig R,Ruat M<br>PubMed Article URL:http://dx.doi.org/null
Not Applicable / Not Cited	13-6890 was used in western blot to study the effect of mDab1 on amyloid precursor protein processing<br><br>Neurobiology of aging (Mar 2007; 28: 377)<br>**"Expression of mDab1 promotes the stability and processing of amyloid precursor protein and this effect is counteracted by X11alpha."**<br>Author(s):Parisiadou L,Efthimiopoulos S<br>PubMed Article URL:http://dx.doi.org/10.1016/j.neurobiolaging.2005.12.015
Not Applicable / Not Cited	13-6890 was used in western blot to identify a synaptic vesicle protein in which trafficking to synaptic vesicles is regulated by AP-3<br><br>The Journal of biological chemistry (Jun 2004; 279: 25430)<br>**"AP-3-dependent mechanisms control the targeting of a chloride channel (ClC-3) in neuronal and non-neuronal cells."**<br>Author(s):Salazar G,Love R,Styers ML,Werner E,Peden A,Rodriguez S,Gearing M,Wainer BH,Faundez V<br>PubMed Article URL:http://dx.doi.org/10.1074/jbc.M402331200
Not Applicable / Not Cited	13-6890 was used in immunocytochemistry and western blot to investigate the cellular function of signal recognition particle<br><br>Experimental cell research (Feb 2007; 313: 834)<br>**"Inefficient targeting to the endoplasmic reticulum by the signal recognition particle elicits selective defects in post-ER membrane trafficking."**<br>Author(s):Lakkaraju AK,Luyet PP,Parone P,Falguières T,Strub K<br>PubMed Article URL:http://dx.doi.org/10.1016/j.yexcr.2006.12.003
Hamster / Not Cited	136890 was used in western blot to report that activation of GABAB receptors results in a decrease in potassium-chloride cotransporter protein function<br><br>The Journal of neuroscience : the official journal of the Society for Neuroscience (May 2017; 37: 5447)<br>**" Receptor Protein Complex."**<br>Author(s):Wright R,Newey SE,Ilie A,Wefelmeyer W,Raimondo JV,Ginham R,McIlhinney RAJ,Akerman CJ<br>PubMed Article URL:http://dx.doi.org/10.1523/JNEUROSCI.2164-16.2017

For Research Use Only. Not for use in diagnostic procedures. Not for resale without express authorization.

Products are warranted to operate or perform substantially in conformance with published Product specifications in effect at the time of sale, as set forth in the Production documentation, specifications and/or accompanying package inserts ("Documentation"). No claim of suitability for use in applications regulated by FDA is made. The warranty provided herein is valid only when used by properly trained individuals. Unless otherwise stated in the Documentation, this warranty is limited to one year from date of shipment when the Product is subjected to normal, proper and intended usage. This warranty does not extend to anyone other than the Buyer. Any model or sample furnished to Buyer is merely illustrative of the general type and quality of goods and does not represent that any Product will conform to such model or sample.

NO OTHER WARRANTIES, EXPRESS OR IMPLED, ARE GRANTED INCLUDING WITHOUT LIMITATION, IMPLIED WARRANTIES OF MERCHANTABILITY, FITNESS FOR ANY PARTICULAR PURPOSE, OR NON INFRINGEMENT. BUYER'S EXCLUSIVE REMEDY FOR NON-CONFORMING PRODUCTS DURING THE WARRANTY PERIOD IS LIMITED TO REPAIR, REPLACEMENT OF OR REFUND FOR THE NON-CONFORMING PRODUCT(S) AT SELLER'S SOLE OPTION. THERE IS NO OBLIGATION TO REPAIR, REPLACE OR REFUND FOR PRODUCTS AS THE RESULT OF (I) ACCIDENT, DISASTER OR EVENT OF FORCE MAJEURE, (II) MISUSE, FAULT OR NEGLIGENCE OF OR BY BUYER, (III) USE OF THE PRODUCTS IN A MANNER FOR WHICH THEY WERE NOT DESIGNED, OR (IV) IMPROPER STORAGE AND HANDLING OF THE PRODUCTS. Unless otherwise expressly stated on the Product or in the documentation accompanying the Product, the Product is intended for research only and is not to be used for any other purpose, including without limitation, unauthorized commercial uses, in vitro diagnostic uses, ex vivo or in vivo therapeutic uses, or any type of consumption by or application to human or animals.

Human / 1:500	13-6890 was used in western blot to examine the raft and cytoskeletal proteins from intrauterine growth restriction and preeclampsia.<br><br>The Journal of membrane biology (Jun 2011; 241: 127)<br>**"Lipid rafts and cytoskeletal proteins in placental microvilli membranes from preeclamptic and IUGR pregnancies."**<br>Author(s):Riquelme G,Vallejos C,de Gregorio N,Morales B,Godoy V,Berrios M,Bastías N,Rodríguez C<br>PubMed Article URL:http://dx.doi.org/10.1007/s00232-011-9369-3
Not Applicable / Not Cited	13-6890 was used in western blot to investigate oligomerization of solute carrier family 30 member 3/zinc transporter 3<br><br>PloS one (Jun 2009; 4: null)<br>**"SLC30A3 (ZnT3) oligomerization by dityrosine bonds regulates its subcellular localization and metal transport capacity."**<br>Author(s):Salazar G,Falcon-Perez JM,Harrison R,Faundez V<br>PubMed Article URL:http://dx.doi.org/10.1371/journal.pone.0005896
Not Applicable / Not Cited	13-6890 was used in western blot to characterize oligodendroglial SNAREs and their trafficking pathways<br><br>Journal of neuroscience research (Jun 2009; 87: 1760)<br>**"Comprehensive analysis of expression, subcellular localization, and cognate pairing of SNARE proteins in oligodendrocytes."**<br>Author(s):Feldmann A,Winterstein C,White R,Trotter J,Krämer-Albers EM<br>PubMed Article URL:http://dx.doi.org/10.1002/jnr.22020
Not Applicable / Not Cited	13-6890 was used in western blot to characterize the molecular and functional interaction between metabotropic glutamate receptor type 1alpha and caveolin-1 or caveolin-2beta<br><br>Experimental cell research (Oct 2004; 300: 23)<br>**"Mutual regulation between metabotropic glutamate type 1alpha receptor and caveolin proteins: from traffick to constitutive activity."**<br>Author(s):Burgueño J,Canela EI,Mallol J,Lluis C,Franco R,Ciruela F<br>PubMed Article URL:http://dx.doi.org/10.1016/j.yexcr.2004.06.013
Human / Not Cited	13-6890 was used in western blot to study the behavior of the HIV-1 Vpu alleles in the plasma of elite controller patients<br><br>PloS one (Feb 2016; 10: null)<br>**"Modest attenuation of HIV-1 Vpu alleles derived from elite controller plasma."**<br>Author(s):Chen J,Tibroni N,Sauter D,Galaski J,Miura T,Alter G,Mueller B,Haller C,Walker BD,Kirchhoff F,Brumme ZL,Ueno T,Fackler OT<br>PubMed Article URL:http://dx.doi.org/10.1371/journal.pone.0120434
Not Applicable / 1:2000	13-6890 was used in western blot to determine modulation of glutamatergic transmission by Lrp4 in astrocytes<br><br>Nature neuroscience (Aug 2016; 19: 1010)<br>**"Lrp4 in astrocytes modulates glutamatergic transmission."**<br>Author(s):Sun XD,Li L,Liu F,Huang ZH,Bean JC,Jiao HF,Barik A,Kim SM,Wu H,Shen C,Tian Y,Lin TW,Bates R,Sathyamurthy A,Chen YJ,Yin DM,Xiong L,Lin HP,Hu JX,Li BM,Gao TM,Xiong WC,Mei L<br>PubMed Article URL:http://dx.doi.org/10.1038/nn.4326
Not Applicable / Not Cited	13-6890 was used in western blot to find that the epithelial Ca(2+) channel TRPV5 is constitutively internalized in a dynamin- and clathrin-dependent manner and constitutively recycles back to the surface<br><br>The Journal of biological chemistry (Feb 2008; 283: 4077)<br>**"TRPV5 is internalized via clathrin-dependent endocytosis to enter a Ca2+-controlled recycling pathway."**<br>Author(s):van de Graaf SF,Rescher U,Hoenderop JG,Verkaart S,Bindels RJ,Gerke V<br>PubMed Article URL:http://dx.doi.org/10.1074/jbc.M706959200
Not Applicable / Not Cited	13-6890 was used in western blot to report that US2 and US11 from human cytomegalovirus target unassembled heavy chains for degradation<br><br>Molecular immunology (Mar 2006; 43: 1258)<br>**"Human cytomegalovirus-encoded US2 and US11 target unassembled MHC class I heavy chains for degradation."**<br>Author(s):Barel MT,Hassink GC,van Voorden S,Wiertz EJ<br>PubMed Article URL:http://dx.doi.org/10.1016/j.molimm.2005.07.005
Not Applicable / Not Cited	13-6890 was used in western blot to investigate the link between the ERK/MAP kinase pathway and fibronectin in melanoma<br><br>The Journal of investigative dermatology (Feb 2007; 127: 400)<br>**"Tumor-derived fibronectin is involved in melanoma cell invasion and regulated by V600E B-Raf signaling pathway."**<br>Author(s):Gaggioli C,Robert G,Bertolotto C,Bailet O,Abbe P,Spadafora A,Bahadoran P,Ortonne JP,Baron V,Ballotti R,Tartare-Deckert S<br>PubMed Article URL:http://dx.doi.org/10.1038/sj.jid.5700524

For Research Use Only. Not for use in diagnostic procedures. Not for resale without express authorization.

Products are warranted to operate or perform substantially in conformance with published Product specifications in effect at the time of sale, as set forth in the Production documentation, specifications and/or accompanying package inserts ("Documentation"). No claim of suitability for use in applications regulated by FDA is made. The warranty provided herein is valid only when used by properly trained individuals. Unless otherwise stated in the Documentation, this warranty is limited to one year from date of shipment when the Product is subjected to normal, proper and intended usage. This warranty does not extend to anyone other than the Buyer. Any model or sample furnished to Buyer is merely illustrative of the general type and quality of goods and does not represent that any Product will conform to such model or sample.

NO OTHER WARRANTIES, EXPRESS OR IMPLED, ARE GRANTED INCLUDING WITHOUT LIMITATION, IMPLIED WARRANTIES OF MERCHANTABILITY, FITNESS FOR ANY PARTICULAR PURPOSE, OR NON INFRINGEMENT. BUYER'S EXCLUSIVE REMEDY FOR NON-CONFORMING PRODUCTS DURING THE WARRANTY PERIOD IS LIMITED TO REPAIR, REPLACEMENT OF OR REFUND FOR THE NON-CONFORMING PRODUCT(S) AT SELLER'S SOLE OPTION. THERE IS NO OBLIGATION TO REPAIR, REPLACE OR REFUND FOR PRODUCTS AS THE RESULT OF (I) ACCIDENT, DISASTER OR EVENT OF FORCE MAJEURE, (II) MISUSE, FAULT OR NEGLIGENCE OF OR BY BUYER, (III) USE OF THE PRODUCTS IN A MANNER FOR WHICH THEY WERE NOT DESIGNED, OR (IV) IMPROPER STORAGE AND HANDLING OF THE PRODUCTS. Unless otherwise expressly stated on the Product or in the documentation accompanying the Product, the Product is intended for research only and is not to be used for any other purpose, including without limitation, unauthorized commercial uses, in vitro diagnostic uses, ex vivo or in vivo therapeutic uses, or any type of consumption by or application to human or animals.

Human / 1 µg/ml	13-6890 was used in western blot to elucidate the mechanisms by which cholesterol regulates LRP-1 levels and function at the plasma membrane.<br><br>FASEB journal : official publication of the Federation of American Societies for Experimental Biology (Aug 2011; 25: 2770)<br>**"Cell cholesterol modulates metalloproteinase-dependent shedding of low-density lipoprotein receptor-related protein-1 (LRP-1) and clearance function."**<br>Author(s):Selvais C,D'Auria L,Tyteca D,Perrot G,Lemoine P,Troeberg L,Dedieu S,Noël A,Nagase H,Henriet P,Courtoy PJ, Marbaix E,Emonard H<br>PubMed Article URL:http://dx.doi.org/10.1096/fj.10-169508
Not Applicable / 1:500	13-6890 was used in western blot to study native cerebellar iFGF14 complexes by proteomic analysis<br><br>Channels (Austin, Tex.) (Jul 2016; 10: 297)<br>**"Proteomic analysis of native cerebellar iFGF14 complexes."**<br>Author(s):Bosch MK,Nerbonne JM,Townsend RR,Miyazaki H,Nukina N,Ornitz DM,Marionneau C<br>PubMed Article URL:http://dx.doi.org/10.1080/19336950.2016.1153203
Human / Not Cited	13-6890 was used in Western Blotting to provide evidence that palmitic acid directly activates TLR2 by inducing heterodimerization with TLR1 in an NADPH oxidase-dependent manner.<br><br>Journal of immunology (Baltimore, Md. : 1950) (Oct 2013; 191: 4337)<br>**"Inflammasome-mediated secretion of IL-1 in human monocytes through TLR2 activation; modulation by dietary fatty acids."**<br>Author(s):Snodgrass RG,Huang S,Choi IW,Rutledge JC,Hwang DH<br>PubMed Article URL:http://dx.doi.org/10.4049/jimmunol.1300298
Human / Not Cited	13-6890 was used in western blot to test if Rab11b affects plasmalemmal expression of Ca(v)1.2.<br><br>American journal of physiology. Cell physiology (May 2011; 300: C1023)<br>**"Small GTPase Rab11b regulates degradation of surface membrane L-type Cav1.2 channels."**<br>Author(s):Best JM,Foell JD,Buss CR,Delisle BP,Balijepalli RC,January CT,Kamp TJ<br>PubMed Article URL:http://dx.doi.org/10.1152/ajpcell.00288.2010
Rat / Not Cited	136890 was used in western blot to determine the in vivo effects of pentobarbital on brain plasma membranes and lipid rafts<br><br>Biochimica et biophysica acta (Nov 2016; 1858: 2603)<br>**" receptors."**<br>Author(s):Sierra-Valdez FJ,Ruiz-Suárez JC,Delint-Ramirez I<br>PubMed Article URL:http://dx.doi.org/10.1016/j.bbamem.2016.07.011
Not Applicable / Not Cited	13-6890 was used in western blot to elucidate the molecular basis of protein sorting into exosomes<br><br>Cell biology international (Jan 2009; 33: 36)<br>**"Exosomal sorting of the cytoplasmic domain of bovine leukemia virus TM Env protein."**<br>Author(s):De Gassart A,Trentin B,Martin M,Hocquellet A,Bette-Bobillo P,Mamoun R,Vidal M<br>PubMed Article URL:http://dx.doi.org/10.1016/j.cellbi.2008.10.001
Not Applicable / Not Cited	13-6890 was used in western blot to explore the roles of GGA2 in lysosomal enzyme transport<br><br>Archives of histology and cytology (Dec 2007; 70: 303)<br>**"Specific depletion of GGA2 causes cathepsin D missorting in HeLa cells."**<br>Author(s):Hida T,Ikeda H,Kametaka S,Akazawa C,Kohsaka S,Ebisu S,Uchiyama Y,Waguri S<br>PubMed Article URL:http://dx.doi.org/null
Human / Not Cited	13-6890 was used in western blot to test if ubiquitination regulates the internalization and degradation of bile salt export pump and multidrug resistance-associated protein 2.<br><br>Molecular pharmacology (Mar 2014; 85: 482)<br>**"Differential roles of ubiquitination in the degradation mechanism of cell surface-resident bile salt export pump and multidrug resistance-associated protein 2."**<br>Author(s):Aida K,Hayashi H,Inamura K,Mizuno T,Sugiyama Y<br>PubMed Article URL:http://dx.doi.org/10.1124/mol.113.091090
Human / Not Cited	136890 was used in western blot to elucidate the role of TfR1 in adaptive immunity<br><br>Nature genetics (Jan 2016; 48: 74)<br>**"A missense mutation in TFRC, encoding transferrin receptor 1, causes combined immunodeficiency."**<br>Author(s):Jabara HH,Boyden SE,Chou J,Ramesh N,Massaad MJ,Benson H,Bainter W,Fraulino D,Rahimov F,Sieff C,Liu ZJ, Alshemmari SH,Al-Ramadi BK,Al-Dhekri H,Arnaout R,Abu-Shukair M,Vatsayan A,Silver E,Ahuja S,Davies EG,Sola-Visner M, Ohsumi TK,Andrews NC,Notarangelo LD,Fleming MD,Al-Herz W,Kunkel LM,Geha RS<br>PubMed Article URL:http://dx.doi.org/10.1038/ng.3465

For Research Use Only. Not for use in diagnostic procedures. Not for resale without express authorization.

Products are warranted to operate or perform substantially in conformance with published Product specifications in effect at the time of sale, as set forth in the Production documentation, specifications and/or accompanying package inserts ("Documentation"). No claim of suitability for use in applications regulated by FDA is made. The warranty provided herein is valid only when used by properly trained individuals. Unless otherwise stated in the Documentation, this warranty is limited to one year from date of shipment when the Product is subjected to normal, proper and intended usage. This warranty does not extend to anyone other than the Buyer. Any model or sample furnished to Buyer is merely illustrative of the general type and quality of goods and does not represent that any Product will conform to such model or sample.

NO OTHER WARRANTIES, EXPRESS OR IMPLED, ARE GRANTED INCLUDING WITHOUT LIMITATION, IMPLIED WARRANTIES OF MERCHANTABILITY, FITNESS FOR ANY PARTICULAR PURPOSE, OR NON INFRINGEMENT. BUYER'S EXCLUSIVE REMEDY FOR NON-CONFORMING PRODUCTS DURING THE WARRANTY PERIOD IS LIMITED TO REPAIR, REPLACEMENT OF OR REFUND FOR THE NON-CONFORMING PRODUCT(S) AT SELLER'S SOLE OPTION. THERE IS NO OBLIGATION TO REPAIR, REPLACE OR REFUND FOR PRODUCTS AS THE RESULT OF (I) ACCIDENT, DISASTER OR EVENT OF FORCE MAJEURE, (II) MISUSE, FAULT OR NEGLIGENCE OF OR BY BUYER, (III) USE OF THE PRODUCTS IN A MANNER FOR WHICH THEY WERE NOT DESIGNED, OR (IV) IMPROPER STORAGE AND HANDLING OF THE PRODUCTS. Unless otherwise expressly stated on the Product or in the documentation accompanying the Product, the Product is intended for research only and is not to be used for any other purpose, including without limitation, unauthorized commercial uses, in vitro diagnostic uses, ex vivo or in vivo therapeutic uses, or any type of consumption by or application to human or animals.

Not Applicable / Not Cited	13-6890 was used in western blot to propose that the coupling of zinc transporter 3 and Vglut1 transport mechanisms regulates neurotransmitter content in secretory vesicles<br><br>Journal of cell science (May 2005; 118: 1911)<br>**"Vglut1 and ZnT3 co-targeting mechanisms regulate vesicular zinc stores in PC12 cells."**<br>Author(s):Salazar G,Craige B,Love R,Kalman D,Faundez V<br>PubMed Article URL:http://dx.doi.org/10.1242/jcs.02319
Human / Not Cited	136890 was used in western blot to assess the relationship between duodenal cytochrome b function and breast cancer prognosis<br><br>Breast cancer research : BCR (Mar 2017; 19: null)<br>**"DCYTB is a predictor of outcome in breast cancer that functions via iron-independent mechanisms."**<br>Author(s):Lemler DJ,Lynch ML,Tesfay L,Deng Z,Paul BT,Wang X,Hegde P,Manz DH,Torti SV,Torti FM<br>PubMed Article URL:http://dx.doi.org/10.1186/s13058-017-0814-9
Not Applicable / Not Cited	13-6890 was used in western blot to report a method to isolate complexes associated with integrin adhesion receptors<br><br>Science signaling (Sep 2009; 2: null)<br>**"Proteomic analysis of integrin-associated complexes identifies RCC2 as a dual regulator of Rac1 and Arf6."**<br>Author(s):Humphries JD,Byron A,Bass MD,Craig SE,Pinney JW,Knight D,Humphries MJ<br>PubMed Article URL:http://dx.doi.org/10.1126/scisignal.2000396
Not Applicable / Not Cited	13-6890 was used in western blot to screen for proteins that bound the protease-associated domain of GRAIL<br><br>The Journal of biological chemistry (Oct 2008; 283: 28497)<br>**"The single subunit transmembrane E3 ligase gene related to anergy in lymphocytes (GRAIL) captures and then ubiquitinates transmembrane proteins across the cell membrane."**<br>Author(s):Lineberry N,Su L,Soares L,Fathman CG<br>PubMed Article URL:http://dx.doi.org/10.1074/jbc.M805092200
Not Applicable / 1:500	13-6890 was used in western blot to investigate the expression, localization, and interactions of the P2X(4)R<br><br>The international journal of biochemistry and cell biology (Oct 2008; 40: 2230)<br>**"Characterization of the molecular interaction between caveolin-1 and the P2X receptors 4 and 7 in E10 mouse lung alveolar epithelial cells."**<br>Author(s):Barth K,Weinhold K,Guenther A,Linge A,Gereke M,Kasper M<br>PubMed Article URL:http://dx.doi.org/10.1016/j.biocel.2008.03.001
Human / 1:1000	13-6890 was used in western blot to report a unbiased global view of SNX27-mediated sorting.<br><br>Nature cell biology (May 2013; 15: 461)<br>**"A global analysis of SNX27-retromer assembly and cargo specificity reveals a function in glucose and metal ion transport."**<br>Author(s):Steinberg F,Gallon M,Winfield M,Thomas EC,Bell AJ,Heesom KJ,Tavaré JM,Cullen PJ<br>PubMed Article URL:http://dx.doi.org/10.1038/ncb2721
Not Applicable / Not Cited	13-6890 was used in western blot to examine the role of galectin-5 in the exosomal sorting pathway during rat reticulocyte maturation<br><br>Blood (Jan 2010; 115: 696)<br>**"Galectin-5 is bound onto the surface of rat reticulocyte exosomes and modulates vesicle uptake by macrophages."**<br>Author(s):Barrès C,Blanc L,Bette-Bobillo P,André S,Mamoun R,Gabius HJ,Vidal M<br>PubMed Article URL:http://dx.doi.org/10.1182/blood-2009-07-231449
Hamster / Not Cited	13-6890 was used in western blot elucidate the interactions among Rab4a, Rabip4, and CD2AP<br><br>Methods in enzymology (Apr 2006; 403: 107)<br>**"CD2AP, Rabip4, and Rabip4': analysis of interaction with Rab4a and regulation of endosomes morphology."**<br>Author(s):Monzo P,Mari M,Kaddai V,Gonzalez T,Le Marchand-Brustel Y,Cormont M<br>PubMed Article URL:http://dx.doi.org/10.1016/S0076-6879(05)03010-7
Not Applicable / 1:500	13-6890 was used in western blot to investigate the use of TfR1 by the glycoproteins from a panel of New World clade B arenaviruses<br><br>Journal of virology (Jan 2008; 82: 938)<br>**"New world clade B arenaviruses can use transferrin receptor 1 (TfR1)-dependent and -independent entry pathways, and glycoproteins from human pathogenic strains are associated with the use of TfR1."**<br>Author(s):Flanagan ML,Oldenburg J,Reignier T,Holt N,Hamilton GA,Martin VK,Cannon PM<br>PubMed Article URL:http://dx.doi.org/10.1128/JVI.01397-07

For Research Use Only. Not for use in diagnostic procedures. Not for resale without express authorization.

Products are warranted to operate or perform substantially in conformance with published Product specifications in effect at the time of sale, as set forth in the Production documentation, specifications and/or accompanying package inserts ("Documentation"). No claim of suitability for use in applications regulated by FDA is made. The warranty provided herein is valid only when used by properly trained individuals. Unless otherwise stated in the Documentation, this warranty is limited to one year from date of shipment when the Product is subjected to normal, proper and intended usage. This warranty does not extend to anyone other than the Buyer. Any model or sample furnished to Buyer is merely illustrative of the general type and quality of goods and does not represent that any Product will conform to such model or sample.

NO OTHER WARRANTIES, EXPRESS OR IMPLED, ARE GRANTED INCLUDING WITHOUT LIMITATION, IMPLIED WARRANTIES OF MERCHANTABILITY, FITNESS FOR ANY PARTICULAR PURPOSE, OR NON INFRINGEMENT. BUYER'S EXCLUSIVE REMEDY FOR NON-CONFORMING PRODUCTS DURING THE WARRANTY PERIOD IS LIMITED TO REPAIR, REPLACEMENT OF OR REFUND FOR THE NON-CONFORMING PRODUCT(S) AT SELLER'S SOLE OPTION. THERE IS NO OBLIGATION TO REPAIR, REPLACE OR REFUND FOR PRODUCTS AS THE RESULT OF (I) ACCIDENT, DISASTER OR EVENT OF FORCE MAJEURE, (II) MISUSE, FAULT OR NEGLIGENCE OF OR BY BUYER, (III) USE OF THE PRODUCTS IN A MANNER FOR WHICH THEY WERE NOT DESIGNED, OR (IV) IMPROPER STORAGE AND HANDLING OF THE PRODUCTS. Unless otherwise expressly stated on the Product or in the documentation accompanying the Product, the Product is intended for research only and is not to be used for any other purpose, including without limitation, unauthorized commercial uses, in vitro diagnostic uses, ex vivo or in vivo therapeutic uses, or any type of consumption by or application to human or animals.

Not Applicable / 1:500	13-6890 was used in western blot to determine if Whitewater Arroyo virus glycoprotein uses hTfR1 for entry, and compare its tropism to glycoproteins from non-pathogenic New World clade B arenaviruses<br><br>Virology (Feb 2008; 371: 439)<br>**"Receptor use by the Whitewater Arroyo virus glycoprotein."**<br>Author(s):Reignier T,Oldenburg J,Flanagan ML,Hamilton GA,Martin VK,Cannon PM<br>PubMed Article URL:http://dx.doi.org/10.1016/j.virol.2007.10.004
Human / Not Cited	13-6890 was used in western blot to unravel the functional relation between mitochondria-shaping proteins and the small GTPase Rab11a<br><br>The Journal of biological chemistry (Jan 2014; 289: 2230)<br>**"A functional interplay between the small GTPase Rab11a and mitochondria-shaping proteins regulates mitochondrial positioning and polarization of the actin cytoskeleton downstream of Src family kinases."**<br>Author(s):Landry MC,Champagne C,Boulanger MC,Jetté A,Fuchs M,Dziengelewski C,Lavoie JN<br>PubMed Article URL:http://dx.doi.org/10.1074/jbc.M113.516351
Not Applicable / Not Cited	13-6890 was used in western blot to develop and characterize a murine model to study enterotoxigenic Bacteroides fragilis infection<br><br>Infection and immunity (Apr 2009; 77: 1708)<br>**"Induction of persistent colitis by a human commensal, enterotoxigenic Bacteroides fragilis, in wild-type C57BL/6 mice."**<br>Author(s):Rhee KJ,Wu S,Wu X,Huso DL,Karim B,Franco AA,Rabizadeh S,Golub JE,Mathews LE,Shin J,Sartor RB, Golenbock D,Hamad AR,Gan CM,Housseau F,Sears CL<br>PubMed Article URL:http://dx.doi.org/10.1128/IAI.00814-08
Not Applicable / Not Cited	13-6890 was used in western blot to identify a role for lipid rafts in the modulation of the shedding of the neurotrophin receptor p75<br><br>FEBS letters (May 2007; 581: 1851)<br>**"Shedding of the p75NTR neurotrophin receptor is modulated by lipid rafts."**<br>Author(s):Gil C,Cubí R,Aguilera J<br>PubMed Article URL:http://dx.doi.org/10.1016/j.febslet.2007.03.080
Not Applicable / Not Cited	13-6890 was used in western blot to study how Bacillus anthracis lethal toxin promotes caspase-3 activation and the formation of thick actin cables in human endothelial cells<br><br>Cellular microbiology (Jul 2010; 12: 891)<br>**"Transcriptome dysregulation by anthrax lethal toxin plays a key role in induction of human endothelial cell cytotoxicity."**<br>Author(s):Rolando M,Stefani C,Flatau G,Auberger P,Mettouchi A,Mhlanga M,Rapp U,Galmiche A,Lemichez E<br>PubMed Article URL:http://dx.doi.org/10.1111/j.1462-5822.2010.01438.x
Human / 1:500	13-6890 was used in western blot to elucidate the cellular functions of GPR56 receptor and how they contribute to bilateral frontoparietal polymicrogyria.<br><br>The Journal of biological chemistry (Apr 2011; 286: 14215)<br>**"Disease-associated GPR56 mutations cause bilateral frontoparietal polymicrogyria via multiple mechanisms."**<br>Author(s):Chiang NY,Hsiao CC,Huang YS,Chen HY,Hsieh IJ,Chang GW,Lin HH<br>PubMed Article URL:http://dx.doi.org/10.1074/jbc.M110.183830
Human / 1:2500	13-6890 was used in western blot to investigate the regulation of SAC primes kinetochore phosphatases<br><br>Nature cell biology (Dec 2014; 16: 1257)<br>**"Negative feedback at kinetochores underlies a responsive spindle checkpoint signal."**<br>Author(s):Nijenhuis W,Vallardi G,Teixeira A,Kops GJ,Saurin AT<br>PubMed Article URL:http://dx.doi.org/10.1038/ncb3065
Human / Not Cited	13-6890 was used in western blot to test if RNF114 modulates RIG-I/MDA5 signaling.<br><br>Human molecular genetics (Aug 2011; 20: 3129)<br>**"Functional analysis of the RNF114 psoriasis susceptibility gene implicates innate immune responses to double-stranded RNA in disease pathogenesis."**<br>Author(s):Bijlmakers MJ,Kanneganti SK,Barker JN,Trembath RC,Capon F<br>PubMed Article URL:http://dx.doi.org/10.1093/hmg/ddr215

For Research Use Only. Not for use in diagnostic procedures. Not for resale without express authorization.

Products are warranted to operate or perform substantially in conformance with published Product specifications in effect at the time of sale, as set forth in the Production documentation, specifications and/or accompanying package inserts ("Documentation"). No claim of suitability for use in applications regulated by FDA is made. The warranty provided herein is valid only when used by properly trained individuals. Unless otherwise stated in the Documentation, this warranty is limited to one year from date of shipment when the Product is subjected to normal, proper and intended usage. This warranty does not extend to anyone other than the Buyer. Any model or sample furnished to Buyer is merely illustrative of the general type and quality of goods and does not represent that any Product will conform to such model or sample.

NO OTHER WARRANTIES, EXPRESS OR IMPLED, ARE GRANTED INCLUDING WITHOUT LIMITATION, IMPLIED WARRANTIES OF MERCHANTABILITY, FITNESS FOR ANY PARTICULAR PURPOSE, OR NON INFRINGEMENT. BUYER'S EXCLUSIVE REMEDY FOR NON-CONFORMING PRODUCTS DURING THE WARRANTY PERIOD IS LIMITED TO REPAIR, REPLACEMENT OF OR REFUND FOR THE NON-CONFORMING PRODUCT(S) AT SELLER'S SOLE OPTION. THERE IS NO OBLIGATION TO REPAIR, REPLACE OR REFUND FOR PRODUCTS AS THE RESULT OF (I) ACCIDENT, DISASTER OR EVENT OF FORCE MAJEURE, (II) MISUSE, FAULT OR NEGLIGENCE OF OR BY BUYER, (III) USE OF THE PRODUCTS IN A MANNER FOR WHICH THEY WERE NOT DESIGNED, OR (IV) IMPROPER STORAGE AND HANDLING OF THE PRODUCTS. Unless otherwise expressly stated on the Product or in the documentation accompanying the Product, the Product is intended for research only and is not to be used for any other purpose, including without limitation, unauthorized commercial uses, in vitro diagnostic uses, ex vivo or in vivo therapeutic uses, or any type of consumption by or application to human or animals.

Not Applicable / Not Cited	13-6890 was used in western blot to investigate the effect of EGFR ligands on receptor internalization and endocytic sorting<br><br>Traffic (Copenhagen, Denmark) (Aug 2009; 10: 1115)<br>**"Differential effects of EGFR ligands on endocytic sorting of the receptor."**<br>Author(s):Roepstorff K,Grandal MV,Henriksen L,Knudsen SL,Lerdrup M,Grøvdal L,Willumsen BM,van Deurs B<br>PubMed Article URL:http://dx.doi.org/10.1111/j.1600-0854.2009.00943.x
Not Applicable / Not Cited	13-6890 was used in western blot to explore the importance of sterol specificity for the structure and function of caveolae<br><br>The Journal of biological chemistry (May 2008; 283: 14610)<br>**"Cholesterol substitution increases the structural heterogeneity of caveolae."**<br>Author(s):Jansen M,Pietiaïnen VM,Pölönen H,Rasilainen L,Koivusalo M,Ruotsalainen U,Jokitalo E,Ikonen E<br>PubMed Article URL:http://dx.doi.org/10.1074/jbc.M710355200

28 Immunocytochemistry References

Species / Dilution	Summary
Not Applicable / Not Cited	13-6890 was used in immunocytochemistry to report that foot-and-mouth disease virus infection proceeds via clathrin-dependent endocytosis<br><br>Journal of virology (Jul 2005; 79: 8519)<br>**"Early events in integrin alphavbeta6-mediated cell entry of foot-and-mouth disease virus."**<br>Author(s):Berryman S,Clark S,Monaghan P,Jackson T<br>PubMed Article URL:http://dx.doi.org/10.1128/JVI.79.13.8519-8534.2005
Not Applicable / Not Cited	13-6890 was used in immunocytochemistry to study the biochemical properties and functions of human RME-8 (hRME-8) in endocytic pathways<br><br>Cell structure and function (Jun 2008; 33: 35)<br>**"Human RME-8 is involved in membrane trafficking through early endosomes."**<br>Author(s):Fujibayashi A,Taguchi T,Misaki R,Ohtani M,Dohmae N,Takio K,Yamada M,Gu J,Yamakami M,Fukuda M,Waguri S,Uchiyama Y,Yoshimori T,Sekiguchi K<br>PubMed Article URL:http://dx.doi.org/null
Human / 1:500	13-6890 was used in immunocytochemistry to investigate the subcellular trafficking and recycle of CD22<br><br>Traffic (Copenhagen, Denmark) (Mar 2014; 15: 255)<br>**"DropArray™, a wall-less 96-well plate for uptake and immunofluorescence microscopy, confirms CD22 recycles."**<br>Author(s):Ingle GS,Scales SJ<br>PubMed Article URL:http://dx.doi.org/10.1111/tra.12144
Not Applicable / 1:200	13-6890 was used in immunocytochemistry to examine major cytoskeletal elements involved in cellular trafficking of complexes made with PEI derivatives<br><br>Journal of controlled release : official journal of the Controlled Release Society (Sep 2007; 122: 111)<br>**"Cytoskeletal involvement in the cellular trafficking of plasmid/PEI derivative complexes."**<br>Author(s):Grosse S,Aron Y,Thévenot G,Monsigny M,Fajac I<br>PubMed Article URL:http://dx.doi.org/10.1016/j.jconrel.2007.06.015
Not Applicable / 1:200	13-6890 was used in immunocytochemistry to report that the transferrin receptor is a receptor for mink enteritis virus, but not Aleutian mink disease parvovirus<br><br>Virology (Sep 2005; 340: 1)<br>**"Two mink parvoviruses use different cellular receptors for entry into CRFK cells."**<br>Author(s):Park GS,Best SM,Bloom ME<br>PubMed Article URL:http://dx.doi.org/10.1016/j.virol.2005.06.038
Not Applicable / 1:200	13-6890 was used in immunocytochemistry to discuss methods to transfer genes into airway epithelial cells<br><br>Journal of controlled release : official journal of the Controlled Release Society (Jun 2004; 97: 371)<br>**"Recombinant Escherichia coli as a gene delivery vector into airway epithelial cells."**<br>Author(s):Fajac I,Grosse S,Collombet JM,Thevenot G,Goussard S,Danel C,Grillot-Courvalin C<br>PubMed Article URL:http://dx.doi.org/10.1016/j.jconrel.2004.03.025
Not Applicable / Not Cited	13-6890 was used in immunocytochemistry to report that AP-3 and AP-1 function independently in sorting tyrosinase from endosomes to the melanosome<br><br>Molecular biology of the cell (Nov 2005; 16: 5356)<br>**"Functions of adaptor protein (AP)-3 and AP-1 in tyrosinase sorting from endosomes to melanosomes."**<br>Author(s):Theos AC,Tenza D,Martina JA,Hurbain I,Peden AA,Sviderskaya EV,Stewart A,Robinson MS,Bennett DC,Cutler DF,Bonifacino JS,Marks MS,Raposo G<br>PubMed Article URL:http://dx.doi.org/10.1091/mbc.e05-07-0626

For Research Use Only. Not for use in diagnostic procedures. Not for resale without express authorization.

Products are warranted to operate or perform substantially in conformance with published Product specifications in effect at the time of sale, as set forth in the Production documentation, specifications and/or accompanying package inserts ("Documentation"). No claim of suitability for use in applications regulated by FDA is made. The warranty provided herein is valid only when used by properly trained individuals. Unless otherwise stated in the Documentation, this warranty is limited to one year from date of shipment when the Product is subjected to normal, proper and intended usage. This warranty does not extend to anyone other than the Buyer. Any model or sample furnished to Buyer is merely illustrative of the general type and quality of goods and does not represent that any Product will conform to such model or sample.

NO OTHER WARRANTIES, EXPRESS OR IMPLED, ARE GRANTED INCLUDING WITHOUT LIMITATION, IMPLIED WARRANTIES OF MERCHANTABILITY, FITNESS FOR ANY PARTICULAR PURPOSE, OR NON INFRINGEMENT. BUYER'S EXCLUSIVE REMEDY FOR NON-CONFORMING PRODUCTS DURING THE WARRANTY PERIOD IS LIMITED TO REPAIR, REPLACEMENT OF OR REFUND FOR THE NON-CONFORMING PRODUCT(S) AT SELLER'S SOLE OPTION. THERE IS NO OBLIGATION TO REPAIR, REPLACE OR REFUND FOR PRODUCTS AS THE RESULT OF (I) ACCIDENT, DISASTER OR EVENT OF FORCE MAJEURE, (II) MISUSE, FAULT OR NEGLIGENCE OF OR BY BUYER, (III) USE OF THE PRODUCTS IN A MANNER FOR WHICH THEY WERE NOT DESIGNED, OR (IV) IMPROPER STORAGE AND HANDLING OF THE PRODUCTS. Unless otherwise expressly stated on the Product or in the documentation accompanying the Product, the Product is intended for research only and is not to be used for any other purpose, including without limitation, unauthorized commercial uses, in vitro diagnostic uses, ex vivo or in vivo therapeutic uses, or any type of consumption by or application to human or animals.

Not Applicable / 1:200	13-6890 was used in immunocytochemistry to describe a point mutant in Rab11 family interacting protein 2 that alters the function of Rab11a-containing trafficking systems<br><br>American journal of physiology. Cell physiology (Sep 2007; 293: C1059)<br>**"Rab11-FIP2 regulates differentiable steps in transcytosis."**<br>Author(s):Ducharme NA,Williams JA,Oztan A,Apodaca G,Lapierre LA,Goldenring JR<br>PubMed Article URL:http://dx.doi.org/10.1152/ajpcell.00078.2007
Not Applicable / 1:200	13-6890 was used in immunocytochemistry to investigate the contribution of the lactose residue on the gene transfer efficiency of lactosylated polyethylenimine<br><br>The journal of gene medicine (Mar 2004; 6: 345)<br>**"Lactosylated polyethylenimine for gene transfer into airway epithelial cells: role of the sugar moiety in cell delivery and intracellular trafficking of the complexes."**<br>Author(s):Grosse S,Aron Y,Honoré I,Thévenot G,Danel C,Roche AC,Monsigny M,Fajac I<br>PubMed Article URL:http://dx.doi.org/10.1002/jgm.515
Not Applicable / Not Cited	13-6890 was used in immunocytochemistry to report that gPr80gag facilitates the release of Moloney murine leukemia virus<br><br>Proceedings of the National Academy of Sciences of the United States of America (Jan 2010; 107: 1190)<br>**"Murine leukemia virus glycosylated Gag (gPr80gag) facilitates interferon-sensitive virus release through lipid rafts."**<br>Author(s):Nitta T,Kuznetsov Y,McPherson A,Fan H<br>PubMed Article URL:http://dx.doi.org/10.1073/pnas.0908660107
Not Applicable / Not Cited	13-6890 was used in immunocytochemistry to study TPC-deficient cells by expression of Ca2+-permeable two-pore channels and rescue of NAADP signaling<br><br>The EMBO journal (Jul 2015; 34: 1743)<br>**"Expression of Ca^{2}-permeable two-pore channels rescues NAADP signalling in TPC-deficient cells."**<br>Author(s):Ruas M,Davis LC,Chen CC,Morgan AJ,Chuang KT,Walseth TF,Grimm C,Garnham C,Powell T,Platt N,Platt FM,Biel M,Wahl-Schott C,Parrington J,Galione A<br>PubMed Article URL:http://dx.doi.org/10.15252/embj.201490009
Not Applicable / Not Cited	13-6890 was used in immunocytochemistry to report how the overexpression of SNX17 influences the endocytic trafficking of P-selectin<br><br>Molecular biology of the cell (Jul 2004; 15: 3095)<br>**"Sorting nexin 17 accelerates internalization yet retards degradation of P-selectin."**<br>Author(s):Williams R,Schlüter T,Roberts MS,Knauth P,Bohnensack R,Cutler DF<br>PubMed Article URL:http://dx.doi.org/10.1091/mbc.e04-02-0143
Not Applicable / Not Cited	13-6890 was used in immunocytochemistry to study recycling endosome transport requires EHD1 recruitment of phosphatidylserine translocase<br><br>The EMBO journal (Mar 2015; 34: 669)<br>**"Transport through recycling endosomes requires EHD1 recruitment by a phosphatidylserine translocase."**<br>Author(s):Lee S,Uchida Y,Wang J,Matsudaira T,Nakagawa T,Kishimoto T,Mukai K,Inaba T,Kobayashi T,Molday RS,Taguchi T,Arai H<br>PubMed Article URL:http://dx.doi.org/10.15252/embj.201489703
Not Applicable / Not Cited	13-6890 was used in immunocytochemistry and western blot to demonstrate that treatment of K562 cells with the phorbol ester TPA induces the down-modulation of various surface antigens<br><br>Journal of cellular biochemistry (Oct 2007; 102: 650)<br>**"The transferrin receptor and the tetraspanin web molecules CD9, CD81, and CD9P-1 are differentially sorted into exosomes after TPA treatment of K562 cells."**<br>Author(s):Abache T,Le Naour F,Planchon S,Harper F,Boucheix C,Rubinstein E<br>PubMed Article URL:http://dx.doi.org/10.1002/jcb.21318
Human / 1:100	13-6890 was used in immunocytochemistry to characterize Kv2.1/Kv6.4/KCNE5 channel complexes<br><br>Scientific reports (Aug 2015; 5: null)<br>**"Auxiliary KCNE subunits modulate both homotetrameric Kv2.1 and heterotetrameric Kv2.1/Kv6.4 channels."**<br>Author(s):David JP,Stas JI,Schmitt N,Bocksteins E<br>PubMed Article URL:http://dx.doi.org/10.1038/srep12813

For Research Use Only. Not for use in diagnostic procedures. Not for resale without express authorization.

Products are warranted to operate or perform substantially in conformance with published Product specifications in effect at the time of sale, as set forth in the Production documentation, specifications and/or accompanying package inserts ("Documentation"). No claim of suitability for use in applications regulated by FDA is made. The warranty provided herein is valid only when used by properly trained individuals. Unless otherwise stated in the Documentation, this warranty is limited to one year from date of shipment when the Product is subjected to normal, proper and intended usage. This warranty does not extend to anyone other than the Buyer. Any model or sample furnished to Buyer is merely illustrative of the general type and quality of goods and does not represent that any Product will conform to such model or sample.

NO OTHER WARRANTIES, EXPRESS OR IMPLED, ARE GRANTED INCLUDING WITHOUT LIMITATION, IMPLIED WARRANTIES OF MERCHANTABILITY, FITNESS FOR ANY PARTICULAR PURPOSE, OR NON INFRINGEMENT. BUYER'S EXCLUSIVE REMEDY FOR NON-CONFORMING PRODUCTS DURING THE WARRANTY PERIOD IS LIMITED TO REPAIR, REPLACEMENT OF OR REFUND FOR THE NON-CONFORMING PRODUCT(S) AT SELLER'S SOLE OPTION. THERE IS NO OBLIGATION TO REPAIR, REPLACE OR REFUND FOR PRODUCTS AS THE RESULT OF (I) ACCIDENT, DISASTER OR EVENT OF FORCE MAJEURE, (II) MISUSE, FAULT OR NEGLIGENCE OF OR BY BUYER, (III) USE OF THE PRODUCTS IN A MANNER FOR WHICH THEY WERE NOT DESIGNED, OR (IV) IMPROPER STORAGE AND HANDLING OF THE PRODUCTS. Unless otherwise expressly stated on the Product or in the documentation accompanying the Product, the Product is intended for research only and is not to be used for any other purpose, including without limitation, unauthorized commercial uses, in vitro diagnostic uses, ex vivo or in vivo therapeutic uses, or any type of consumption by or application to human or animals.

Not Applicable / Not Cited	13-6890 was used in immunocytochemistry to propose that BIG2 regulates the structural integrity of the recycling endosome through activating class I ADP-ribosylation factors<br><br>Molecular biology of the cell (Dec 2004; 15: 5283)<br>**"BIG2, a guanine nucleotide exchange factor for ADP-ribosylation factors: its localization to recycling endosomes and implication in the endosome integrity."**<br>Author(s):Shin HW,Morinaga N,Noda M,Nakayama K<br>PubMed Article URL:http://dx.doi.org/10.1091/mbc.e04-05-0388
Not Applicable / Not Cited	13-6890 was used in immunocytochemistry and western blot to assess if the exocyst complex is a multipurpose regulator of endocytic traffic directed toward both poles of polarized epithelial cells<br><br>Molecular biology of the cell (Oct 2007; 18: 3978)<br>**"Exocyst requirement for endocytic traffic directed toward the apical and basolateral poles of polarized MDCK cells."**<br>Author(s):Oztan A,Silvis M,Weisz OA,Bradbury NA,Hsu SC,Goldenring JR,Yeaman C,Apodaca G<br>PubMed Article URL:http://dx.doi.org/10.1091/mbc.e07-02-0097
Not Applicable / Not Cited	13-6890 was used in immunocytochemistry to test if existing microvesicle pathways transfer RNA genomes horizontally between cells<br><br>PloS one (Jul 2009; 4: null)<br>**"Cellular microvesicle pathways can be targeted to transfer genetic information between non-immune cells."**<br>Author(s):Skinner AM,O'Neill SL,Kurre P<br>PubMed Article URL:http://dx.doi.org/10.1371/journal.pone.0006219
Not Applicable / Not Cited	13-6890 was used in immunocytochemistry to promote biogenesis of membrane-trafficking intermediates by engagement of OCRL1 with F-BAR protein pacsin 2<br><br>Molecular biology of the cell (Jan 2016; 27: 90)<br>**"OCRL1 engages with the F-BAR protein pacsin 2 to promote biogenesis of membrane-trafficking intermediates."**<br>Author(s):Billcliff PG,Noakes CJ,Mehta ZB,Yan G,Mak L,Woscholski R,Lowe M<br>PubMed Article URL:http://dx.doi.org/10.1091/mbc.E15-06-0329
Human / Not Cited	13-6890 was used in immunocytochemistry and western blot to ascertain the role of heterodimerization in zinc transporter sorting in the endolysosomal pathway<br><br>Traffic (Copenhagen, Denmark) (Mar 2016; 17: 267)<br>**"Differential Targeting of SLC30A10/ZnT10 Heterodimers to Endolysosomal Compartments Modulates EGF-Induced MEK/ERK1/2 Activity."**<br>Author(s):Zhao Y,Feresin RG,Falcon-Perez JM,Salazar G<br>PubMed Article URL:http://dx.doi.org/10.1111/tra.12371
Mouse / Not Cited	13-6890 was used in immunocytochemistry to study the role of VAMP3/cellubrevin and VAMP7/TI-VAMP in myelin membrane trafficking.<br><br>The Journal of neuroscience : the official journal of the Society for Neuroscience (Apr 2011; 31: 5659)<br>**"Transport of the major myelin proteolipid protein is directed by VAMP3 and VAMP7."**<br>Author(s):Feldmann A,Amphornrat J,Schönherr M,Winterstein C,Möbius W,Ruhwedel T,Danglot L,Nave KA,Galli T,Bruns D,Trotter J,Krämer-Albers EM<br>PubMed Article URL:http://dx.doi.org/10.1523/JNEUROSCI.6638-10.2011
Not Applicable / Not Cited	13-6890 was used in immunocytochemistry to elucidate mechanisms that regulate ATP7A trafficking<br><br>Molecular biology of the cell (Jun 2013; 24: 1735)<br>**"Trafficking of the Menkes copper transporter ATP7A is regulated by clathrin-, AP-2-, AP-1-, and Rab22-dependent steps."**<br>Author(s):Holloway ZG,Velayos-Baeza A,Howell GJ,Levecque C,Ponnambalam S,Sztul E,Monaco AP<br>PubMed Article URL:http://dx.doi.org/10.1091/mbc.E12-08-0625
Not Applicable / Not Cited	13-6890 was used in immunocytochemistry to determine the effects of Rab5 and RalA on the intracellular trafficking of p-glycoprotein<br><br>Biochimica et biophysica acta (Jul 2007; 1773: 1062)<br>**"The small GTPases Rab5 and RalA regulate intracellular traffic of P-glycoprotein."**<br>Author(s):Fu D,van Dam EM,Brymora A,Duggin IG,Robinson PJ,Roufogalis BD<br>PubMed Article URL:http://dx.doi.org/10.1016/j.bbamcr.2007.03.023

For Research Use Only. Not for use in diagnostic procedures. Not for resale without express authorization.

Products are warranted to operate or perform substantially in conformance with published Product specifications in effect at the time of sale, as set forth in the Production documentation, specifications and/or accompanying package inserts ("Documentation"). No claim of suitability for use in applications regulated by FDA is made. The warranty provided herein is valid only when used by properly trained individuals. Unless otherwise stated in the Documentation, this warranty is limited to one year from date of shipment when the Product is subjected to normal, proper and intended usage. This warranty does not extend to anyone other than the Buyer. Any model or sample furnished to Buyer is merely illustrative of the general type and quality of goods and does not represent that any Product will conform to such model or sample.

NO OTHER WARRANTIES, EXPRESS OR IMPLED, ARE GRANTED INCLUDING WITHOUT LIMITATION, IMPLIED WARRANTIES OF MERCHANTABILITY, FITNESS FOR ANY PARTICULAR PURPOSE, OR NON INFRINGEMENT. BUYER'S EXCLUSIVE REMEDY FOR NON-CONFORMING PRODUCTS DURING THE WARRANTY PERIOD IS LIMITED TO REPAIR, REPLACEMENT OF OR REFUND FOR THE NON-CONFORMING PRODUCT(S) AT SELLER'S SOLE OPTION. THERE IS NO OBLIGATION TO REPAIR, REPLACE OR REFUND FOR PRODUCTS AS THE RESULT OF (I) ACCIDENT, DISASTER OR EVENT OF FORCE MAJEURE, (II) MISUSE, FAULT OR NEGLIGENCE OF OR BY BUYER, (III) USE OF THE PRODUCTS IN A MANNER FOR WHICH THEY WERE NOT DESIGNED, OR (IV) IMPROPER STORAGE AND HANDLING OF THE PRODUCTS. Unless otherwise expressly stated on the Product or in the documentation accompanying the Product, the Product is intended for research only and is not to be used for any other purpose, including without limitation, unauthorized commercial uses, in vitro diagnostic uses, ex vivo or in vivo therapeutic uses, or any type of consumption by or application to human or animals.

Not Applicable / Not Cited	13-6890 was used in immunocytochemistry to investigate the sorting of cargo receptors<br><br>Journal of cell science (Jul 2005; 118: 3003)<br>**"Depletion of TSG101 forms a mammalian "Class E" compartment: a multicisternal early endosome with multiple sorting defects."**<br>Author(s):Doyotte A,Russell MR,Hopkins CR,Woodman PG<br>PubMed Article URL:http://dx.doi.org/10.1242/jcs.02421
Human / Not Cited	13-6890 was used in immunocytochemistry to test if Nef modifies the composition of exosomes released by T lymphocytes<br><br>PloS one (Aug 2015; 9: null)<br>**"Nef neutralizes the ability of exosomes from CD4+ T cells to act as decoys during HIV-1 infection."**<br>Author(s):de Carvalho JV,de Castro RO,da Silva EZ,Silveira PP,da Silva-Januário ME,Arruda E,Jamur MC,Oliver C,Aguiar RS,daSilva LL<br>PubMed Article URL:http://dx.doi.org/10.1371/journal.pone.0113691
Not Applicable / Not Cited	13-6890 was used in immunocytochemistry to identify and study class III rhodopsin mutations<br><br>The Journal of clinical investigation (Jul 2004; 114: 131)<br>**"Structural and functional impairment of endocytic pathways by retinitis pigmentosa mutant rhodopsin-arrestin complexes."**<br>Author(s):Chuang JZ,Vega C,Jun W,Sung CH<br>PubMed Article URL:http://dx.doi.org/10.1172/JCI21136
Pig / 1:25	136890 was used in immunocytochemistry to study the entry and uncoating of African swine fever virus<br><br>PLoS pathogens (Apr 2016; 12: null)<br>**"African Swine Fever Virus Undergoes Outer Envelope Disruption, Capsid Disassembly and Inner Envelope Fusion before Core Release from Multivesicular Endosomes."**<br>Author(s):Hernáez B,Guerra M,Salas ML,Andrés G<br>PubMed Article URL:http://dx.doi.org/10.1371/journal.ppat.1005595
Not Applicable / Not Cited	13-6890 was used in immunocytochemistry to elucidate the mechanisms that regulate the retrieval of the cation-independent mannose 6-phosphate receptor<br><br>The Journal of cell biology (Apr 2004; 165: 123)<br>**"Role of the mammalian retromer in sorting of the cation-independent mannose 6-phosphate receptor."**<br>Author(s):Arighi CN,Hartnell LM,Aguilar RC,Haft CR,Bonifacino JS<br>PubMed Article URL:http://dx.doi.org/10.1083/jcb.200312055

3 Immunohistochemistry (Paraffin) References

Species / Dilution	Summary
Human / 1:2000	136890 was used in immunohistochemistry - paraffin section to use SILAC immunoprecipitation to identify antibody targets expressed by vaccinated cancer patients<br><br>Cancer immunology research (Mar 2016; 4: 225)<br>**"Using Quantitative Seroproteomics to Identify Antibody Biomarkers in Pancreatic Cancer."**<br>Author(s):Jhaveri DT,Kim MS,Thompson ED,Huang L,Sharma R,Klein AP,Zheng L,Le DT,Laheru DA,Pandey A,Jaffee EM,Anders RA<br>PubMed Article URL:http://dx.doi.org/10.1158/2326-6066.CIR-15-0200-T
Not Applicable / 1:200	13-6890 was used in immunohistochemistry - paraffin section to determine the role of metal-related molecules in hepatocarcinogenesis<br><br>Archives of toxicology (Apr 2010; 84: 319)<br>**"Induction of GST-P-positive proliferative lesions facilitating lipid peroxidation with possible involvement of transferrin receptor up-regulation and ceruloplasmin down-regulation from the early stage of liver tumor promotion in rats."**<br>Author(s):Mizukami S,Ichimura R,Kemmochi S,Taniai E,Shimamoto K,Ohishi T,Takahashi M,Mitsumori K,Shibutani M<br>PubMed Article URL:http://dx.doi.org/10.1007/s00204-009-0496-x
Human / 1:100	13-6890 was used in immunohistochemistry - paraffin section and western blot to test if TfR1/CD71 is differentially expressed among benign and malignant thyroid tissues.<br><br>Thyroid : official journal of the American Thyroid Association (Mar 2011; 21: 267)<br>**"Aberrant expression of TfR1/CD71 in thyroid carcinomas identifies a novel potential diagnostic marker and therapeutic target."**<br>Author(s):Magro G,Cataldo I,Amico P,Torrisi A,Vecchio GM,Parenti R,Asioli S,Recupero D,D'Agata V,Mucignat MT,Perris R<br>PubMed Article URL:http://dx.doi.org/10.1089/thy.2010.0173

70 Miscellaneous PubMed References

For Research Use Only. Not for use in diagnostic procedures. Not for resale without express authorization.

Products are warranted to operate or perform substantially in conformance with published Product specifications in effect at the time of sale, as set forth in the Production documentation, specifications and/or accompanying package inserts ("Documentation"). No claim of suitability for use in applications regulated by FDA is made. The warranty provided herein is valid only when used by properly trained individuals. Unless otherwise stated in the Documentation, this warranty is limited to one year from date of shipment when the Product is subjected to normal, proper and intended usage. This warranty does not extend to anyone other than the Buyer. Any model or sample furnished to Buyer is merely illustrative of the general type and quality of goods and does not represent that any Product will conform to such model or sample.

NO OTHER WARRANTIES, EXPRESS OR IMPLED, ARE GRANTED INCLUDING WITHOUT LIMITATION, IMPLIED WARRANTIES OF MERCHANTABILITY, FITNESS FOR ANY PARTICULAR PURPOSE, OR NON INFRINGEMENT. BUYER'S EXCLUSIVE REMEDY FOR NON-CONFORMING PRODUCTS DURING THE WARRANTY PERIOD IS LIMITED TO REPAIR, REPLACEMENT OF OR REFUND FOR THE NON-CONFORMING PRODUCT(S) AT SELLER'S SOLE OPTION. THERE IS NO OBLIGATION TO REPAIR, REPLACE OR REFUND FOR PRODUCTS AS THE RESULT OF (I) ACCIDENT, DISASTER OR EVENT OF FORCE MAJEURE, (II) MISUSE, FAULT OR NEGLIGENCE OF OR BY BUYER, (III) USE OF THE PRODUCTS IN A MANNER FOR WHICH THEY WERE NOT DESIGNED, OR (IV) IMPROPER STORAGE AND HANDLING OF THE PRODUCTS. Unless otherwise expressly stated on the Product or in the documentation accompanying the Product, the Product is intended for research only and is not to be used for any other purpose, including without limitation, unauthorized commercial uses, in vitro diagnostic uses, ex vivo or in vivo therapeutic uses, or any type of consumption by or application to human or animals.

Species / Dilution	Summary
Human / Not Cited	13-6890 was used in western blot to study the use of Tf-CRM107 for brain tumor therapy.<br><br>Cancer research (Jan 2000; 60: 230)<br>**"Vascular protection by chloroquine during brain tumor therapy with Tf-CRM107."**<br>Author(s):Hagihara N,Walbridge S,Olson AW,Oldfield EH,Youle RJ<br>PubMed Article URL:http://dx.doi.org/null
Dog / Not Cited	13-6890 was used in western blot to determine the role of exchange factor for ARF6 during the development of epithelial cell polarity.<br><br>Molecular biology of the cell (Mar 2004; 15: 1134)<br>**"EFA6, exchange factor for ARF6, regulates the actin cytoskeleton and associated tight junction in response to E-cadherin engagement."**<br>Author(s):Luton F,Klein S,Chauvin JP,Le Bivic A,Bourgoin S,Franco M,Chardin P<br>PubMed Article URL:http://dx.doi.org/10.1091/mbc.e03-10-0751
Human / Not Cited	13-6890 was used in immunocytochemistry to describe the membrane organization of the recycling pathway.<br><br>The Journal of cell biology (May 2000; 149: 901)<br>**"Distinct membrane domains on endosomes in the recycling pathway visualized by multicolor imaging of Rab4, Rab5, and Rab11."**<br>Author(s):Sönnichsen B,De Renzis S,Nielsen E,Rietdorf J,Zerial M<br>PubMed Article URL:http://dx.doi.org/null
Rat / Not Cited	13-6890 was used in immunocytochemistry to study brefeldin A-induced membrane tubule formation.<br><br>Molecular biology of the cell (Mar 2000; 11: 941)<br>**"Brefeldin A-dependent membrane tubule formation reconstituted in vitro is driven by a cell cycle-regulated microtubule motor."**<br>Author(s):Robertson AM,Allan VJ<br>PubMed Article URL:http://dx.doi.org/10.1091/mbc.11.3.941
Human / 1:200	13-6890 was used in flow cytometry and immunocytochemistry to study the conversion of phosphatidylinositol 3-phosphate to phosphatidylinositol 4-phosphate at endosomes en route to the plasma membrane<br><br>Nature (Jan 2016; 529: 408)<br>**"A phosphoinositide conversion mechanism for exit from endosomes."**<br>Author(s):Ketel K,Krauss M,Nicot AS,Puchkov D,Wieffer M,Müller R,Subramanian D,Schultz C,Laporte J,Haucke V<br>PubMed Article URL:http://dx.doi.org/10.1038/nature16516
Mouse / 1:1000	13-6890 was used in immunohistochemistry to examine the role of Cdc42 in enterocyte polarization<br><br>The Journal of cell biology (Sep 2015; 210: 1055)<br>**"ATP8B1-mediated spatial organization of Cdc42 signaling maintains singularity during enterocyte polarization."**<br>Author(s):Bruurs LJ,Donker L,Zwakenberg S,Zwartkruis FJ,Begthel H,Knisely AS,Posthuma G,van de Graaf SF,Paulusma CC,Bos JL<br>PubMed Article URL:http://dx.doi.org/10.1083/jcb.201505118
Human / Not Cited	13-6890 was used in immunoprecipitation to describe the structure and function of the putative human orthologue of yeast Hrd1p/Der3p.<br><br>The Journal of biological chemistry (Jan 2004; 279: 3525)<br>**"Human HRD1 is an E3 ubiquitin ligase involved in degradation of proteins from the endoplasmic reticulum."**<br>Author(s):Kikkert M,Doolman R,Dai M,Avner R,Hassink G,van Voorden S,Thanedar S,Roitelman J,Chau V,Wiertz E<br>PubMed Article URL:http://dx.doi.org/10.1074/jbc.M307453200
Human / Not Cited	13-6890 was used in western blot to study the role of NHE6 in endocytosis.<br><br>American journal of physiology. Cell physiology (Dec 2011; 301: C1431)<br>**"Na+/H+ exchanger isoform 6 (NHE6/SLC9A6) is involved in clathrin-dependent endocytosis of transferrin."**<br>Author(s):Xinhan L,Matsushita M,Numaza M,Taguchi A,Mitsui K,Kanazawa H<br>PubMed Article URL:http://dx.doi.org/10.1152/ajpcell.00154.2011
Dog / 1:1000	13-6890 was used in western blot to search for compounds to treat intrahepatic cholestasis<br><br>Biochimica et biophysica acta (Sep 2010; 1801: 1005)<br>**"Short- and medium-chain fatty acids enhance the cell surface expression and transport capacity of the bile salt export pump (BSEP/ABCB11)."**<br>Author(s):Kato T,Hayashi H,Sugiyama Y<br>PubMed Article URL:http://dx.doi.org/10.1016/j.bbalip.2010.04.002

For Research Use Only. Not for use in diagnostic procedures. Not for resale without express authorization.

Products are warranted to operate or perform substantially in conformance with published Product specifications in effect at the time of sale, as set forth in the Production documentation, specifications and/or accompanying package inserts ("Documentation"). No claim of suitability for use in applications regulated by FDA is made. The warranty provided herein is valid only when used by properly trained individuals. Unless otherwise stated in the Documentation, this warranty is limited to one year from date of shipment when the Product is subjected to normal, proper and intended usage. This warranty does not extend to anyone other than the Buyer. Any model or sample furnished to Buyer is merely illustrative of the general type and quality of goods and does not represent that any Product will conform to such model or sample.

NO OTHER WARRANTIES, EXPRESS OR IMPLED, ARE GRANTED INCLUDING WITHOUT LIMITATION, IMPLIED WARRANTIES OF MERCHANTABILITY, FITNESS FOR ANY PARTICULAR PURPOSE, OR NON INFRINGEMENT. BUYER'S EXCLUSIVE REMEDY FOR NON-CONFORMING PRODUCTS DURING THE WARRANTY PERIOD IS LIMITED TO REPAIR, REPLACEMENT OF OR REFUND FOR THE NON-CONFORMING PRODUCT(S) AT SELLER'S SOLE OPTION. THERE IS NO OBLIGATION TO REPAIR, REPLACE OR REFUND FOR PRODUCTS AS THE RESULT OF (I) ACCIDENT, DISASTER OR EVENT OF FORCE MAJEURE, (II) MISUSE, FAULT OR NEGLIGENCE OF OR BY BUYER, (III) USE OF THE PRODUCTS IN A MANNER FOR WHICH THEY WERE NOT DESIGNED, OR (IV) IMPROPER STORAGE AND HANDLING OF THE PRODUCTS. Unless otherwise expressly stated on the Product or in the documentation accompanying the Product, the Product is intended for research only and is not to be used for any other purpose, including without limitation, unauthorized commercial uses, in vitro diagnostic uses, ex vivo or in vivo therapeutic uses, or any type of consumption by or application to human or animals.

Human / Not Cited	13-6890 was used in western blot to report a role for a syndecan-4-regulated integrin endocytic pathway in wound repair.<br><br>Developmental cell (Oct 2011; 21: 681)<br>**"A syndecan-4 hair trigger initiates wound healing through caveolin- and RhoG-regulated integrin endocytosis."**<br>Author(s):Bass MD,Williamson RC,Nunan RD,Humphries JD,Byron A,Morgan MR,Martin P,Humphries MJ<br>PubMed Article URL:http://dx.doi.org/10.1016/j.devcel.2011.08.007
Human / Not Cited	13-6890 was used in western blot to identify ZF21 as a regulator of focal adhesion and cell movement<br><br>The Journal of biological chemistry (Jul 2010; 285: 21013)<br>**"ZF21 protein regulates cell adhesion and motility."**<br>Author(s):Nagano M,Hoshino D,Sakamoto T,Kawasaki N,Koshikawa N,Seiki M<br>PubMed Article URL:http://dx.doi.org/10.1074/jbc.M110.106443
Human / Not Cited	13-6890 was used in western blot to discuss methods to isolate fetal cells from the mother.<br><br>Clinical chemistry (Sep 1999; 45: 1614)<br>**"Development, characterization, and use of monoclonal antibodies made to antigens expressed on the surface of fetal nucleated red blood cells."**<br>Author(s):Alvarez FV,Olander J,Crimmins D,Prieto B,Paz A,Alonso R,Porter S,Hess J,Crist RD,Landt Y,Ladenson JH<br>PubMed Article URL:http://dx.doi.org/null
Mouse / Not Cited	13-6890 was used in western blot to test if pharmacological modulation of SPPL2a can deplete B cells.<br><br>Molecular and cellular biology (Apr 2014; 34: 1398)<br>**"The intramembrane proteases signal Peptide peptidase-like 2a and 2b have distinct functions in vivo."**<br>Author(s):Schneppenheim J,Hüttl S,Mentrup T,Lüllmann-Rauch R,Rothaug M,Engelke M,Dittmann K,Dressel R,Araki M,Araki K,Wienands J,Fluhrer R,Saftig P,Schröder B<br>PubMed Article URL:http://dx.doi.org/10.1128/MCB.00038-14
Human / Not Cited	13-6890 was used in immunocytochemistry to determine the cellular distribution and trafficking of a human cytomegalovirus chemokine receptor.<br><br>Molecular biology of the cell (Jun 2001; 12: 1737)<br>**"The human cytomegalovirus US28 protein is located in endocytic vesicles and undergoes constitutive endocytosis and recycling."**<br>Author(s):Fraile-Ramos A,Kledal TN,Pelchen-Matthews A,Bowers K,Schwartz TW,Marsh M<br>PubMed Article URL:http://dx.doi.org/10.1091/mbc.12.6.1737
Hamster / 1 µg/ml	13-6890 was used in western blot to report a molecular and functional association of mGlu1alpha receptor with caveolins.<br><br>Journal of neurochemistry (Aug 2003; 86: 785)<br>**"Metabotropic glutamate type 1alpha receptor localizes in low-density caveolin-rich plasma membrane fractions."**<br>Author(s):Burgueño J,Enrich C,Canela EI,Mallol J,Lluis C,Franco R,Ciruela F<br>PubMed Article URL:http://dx.doi.org/null
Mouse / Not Cited	13-6890 was used in immunocytochemistry to elucidate the role of Arf4 in ciliary protein trafficking.<br><br>PLoS genetics (Feb 2014; 10: null)<br>**"Arf4 is required for Mammalian development but dispensable for ciliary assembly."**<br>Author(s):Follit JA,San Agustin JT,Jonassen JA,Huang T,Rivera-Perez JA,Tremblay KD,Pazour GJ<br>PubMed Article URL:http://dx.doi.org/10.1371/journal.pgen.1004170
Human / Not Cited	13-6890 was used in western blot to investigate transmembrane domains-mediated events during HIV infection.<br><br>Journal of virology (Jan 2012; 86: 757)<br>**"Transmembrane domain determinants of CD4 Downregulation by HIV-1 Vpu."**<br>Author(s):Magadán JG,Bonifacino JS<br>PubMed Article URL:http://dx.doi.org/10.1128/JVI.05933-11
Human / Not Cited	13-6890 was used in immunocytochemistry and western blot to determine the localization and function of Rab15.<br><br>The Journal of biological chemistry (Aug 1999; 274: 22303)<br>**"Rab15 mediates an early endocytic event in Chinese hamster ovary cells."**<br>Author(s):Zuk PA,Elferink LA<br>PubMed Article URL:http://dx.doi.org/null

For Research Use Only. Not for use in diagnostic procedures. Not for resale without express authorization.

Products are warranted to operate or perform substantially in conformance with published Product specifications in effect at the time of sale, as set forth in the Production documentation, specifications and/or accompanying package inserts ("Documentation"). No claim of suitability for use in applications regulated by FDA is made. The warranty provided herein is valid only when used by properly trained individuals. Unless otherwise stated in the Documentation, this warranty is limited to one year from date of shipment when the Product is subjected to normal, proper and intended usage. This warranty does not extend to anyone other than the Buyer. Any model or sample furnished to Buyer is merely illustrative of the general type and quality of goods and does not represent that any Product will conform to such model or sample.

NO OTHER WARRANTIES, EXPRESS OR IMPLED, ARE GRANTED INCLUDING WITHOUT LIMITATION, IMPLIED WARRANTIES OF MERCHANTABILITY, FITNESS FOR ANY PARTICULAR PURPOSE, OR NON INFRINGEMENT. BUYER'S EXCLUSIVE REMEDY FOR NON-CONFORMING PRODUCTS DURING THE WARRANTY PERIOD IS LIMITED TO REPAIR, REPLACEMENT OF OR REFUND FOR THE NON-CONFORMING PRODUCT(S) AT SELLER'S SOLE OPTION. THERE IS NO OBLIGATION TO REPAIR, REPLACE OR REFUND FOR PRODUCTS AS THE RESULT OF (I) ACCIDENT, DISASTER OR EVENT OF FORCE MAJEURE, (II) MISUSE, FAULT OR NEGLIGENCE OF OR BY BUYER, (III) USE OF THE PRODUCTS IN A MANNER FOR WHICH THEY WERE NOT DESIGNED, OR (IV) IMPROPER STORAGE AND HANDLING OF THE PRODUCTS. Unless otherwise expressly stated on the Product or in the documentation accompanying the Product, the Product is intended for research only and is not to be used for any other purpose, including without limitation, unauthorized commercial uses, in vitro diagnostic uses, ex vivo or in vivo therapeutic uses, or any type of consumption by or application to human or animals.

Mouse / Not Cited	13-6890 was used in western blot to describe methods for the assignment of immunoreactive spots in two-dimensional protein patterns.<br><br>Analytical biochemistry (Sep 2002; 308: 381)<br>**"A colloidal silver staining--destaining method for precise assignment of immunoreactive spots in two-dimensional protein patterns."**<br>Author(s):Vettermann C,Jäck HM,Mielenz D<br>PubMed Article URL:http://dx.doi.org/null
Human / 1:50	13-6890 was used in immunohistochemistry - paraffin section and western blot to determine transferrin receptor 1 expression in canine B-cell and T-cell lymphoma<br><br>Veterinary pathology (Mar 2011; 48: 466)<br>**"Transferrin receptor expression in canine lymphoma."**<br>Author(s):Priest H,McDonough S,Erb H,Daddona J,Stokol T<br>PubMed Article URL:http://dx.doi.org/10.1177/0300985810377074
Mouse / Not Cited	13-6890 was used in immunocytochemistry to determine the localization of Afipia felis in infected macrophages.<br><br>Proceedings of the National Academy of Sciences of the United States of America (Jun 2001; 98: 7271)<br>**"Afipia felis induces uptake by macrophages directly into a nonendocytic compartment."**<br>Author(s):Luhrmann A,Streker K,Schüttfort A,Daniels JJ,Haas A<br>PubMed Article URL:http://dx.doi.org/10.1073/pnas.121190398
Rat / Not Cited	13-6890 was used in western blot to elucidate the release of gliotransmitters.<br><br>The Journal of neuroscience : the official journal of the Society for Neuroscience (Feb 2013; 33: 3413)<br>**"Storage and uptake of D-serine into astrocytic synaptic-like vesicles specify gliotransmission."**<br>Author(s):Martineau M,Shi T,Puyal J,Knolhoff AM,Dulong J,Gasnier B,Klingauf J,Sweedler JV,Jahn R,Mothet JP<br>PubMed Article URL:http://dx.doi.org/10.1523/JNEUROSCI.3497-12.2013
Human / Not Cited	13-6890 was used in western blot to determine the occurrence and distribution pattern of uPAR in the rat molar tooth germ.<br><br>Histochemistry and cell biology (Dec 2013; 140: 649)<br>**"Immunocytochemical and biochemical detection of the urokinase-type plasminogen activator receptor (uPAR) in the rat tooth germ and in lipid rafts of PMA-stimulated dental epithelial cells."**<br>Author(s):von Germar A,Barth K,Schwab W<br>PubMed Article URL:http://dx.doi.org/10.1007/s00418-013-1109-6
Rat / Not Cited	13-6890 was used in western blot to study specific sorting of proteins in exosomal membranes.<br><br>Blood (Dec 2003; 102: 4336)<br>**"Lipid raft-associated protein sorting in exosomes."**<br>Author(s):de Gassart A,Geminard C,Fevrier B,Raposo G,Vidal M<br>PubMed Article URL:http://dx.doi.org/10.1182/blood-2003-03-0871
Mouse / Not Cited	13-6890 was used in immunocytochemistry to study L1 endocytosis.<br><br>The Journal of neuroscience : the official journal of the Society for Neuroscience (Jul 1998; 18: 5311)<br>**"The neural cell adhesion molecule L1 interacts with the AP-2 adaptor and is endocytosed via the clathrin-mediated pathway."**<br>Author(s):Kamiguchi H,Long KE,Pendergast M,Schaefer AW,Rapoport I,Kirchhausen T,Lemmon V<br>PubMed Article URL:http://dx.doi.org/null
Rat / Not Cited	13-6890 was used in western blot to examine lipid rafts in the dendrites of cultured hippocampal neurons.<br><br>The Journal of neuroscience : the official journal of the Society for Neuroscience (Apr 2003; 23: 3262)<br>**"Lipid rafts in the maintenance of synapses, dendritic spines, and surface AMPA receptor stability."**<br>Author(s):Hering H,Lin CC,Sheng M<br>PubMed Article URL:http://dx.doi.org/null
Human / Not Cited	13-6890 was used in western blot to study the localization of the transferrin receptor during the formation of the immunological synapse.<br><br>Journal of immunology (Baltimore, Md. : 1950) (Jun 2004; 172: 6709)<br>**"Recruitment of transferrin receptor to immunological synapse in response to TCR engagement."**<br>Author(s):Batista A,Millán J,Mittelbrunn M,Sánchez-Madrid F,Alonso MA<br>PubMed Article URL:http://dx.doi.org/null

For Research Use Only. Not for use in diagnostic procedures. Not for resale without express authorization.

Products are warranted to operate or perform substantially in conformance with published Product specifications in effect at the time of sale, as set forth in the Production documentation, specifications and/or accompanying package inserts ("Documentation"). No claim of suitability for use in applications regulated by FDA is made. The warranty provided herein is valid only when used by properly trained individuals. Unless otherwise stated in the Documentation, this warranty is limited to one year from date of shipment when the Product is subjected to normal, proper and intended usage. This warranty does not extend to anyone other than the Buyer. Any model or sample furnished to Buyer is merely illustrative of the general type and quality of goods and does not represent that any Product will conform to such model or sample.

NO OTHER WARRANTIES, EXPRESS OR IMPLED, ARE GRANTED INCLUDING WITHOUT LIMITATION, IMPLIED WARRANTIES OF MERCHANTABILITY, FITNESS FOR ANY PARTICULAR PURPOSE, OR NON INFRINGEMENT. BUYER'S EXCLUSIVE REMEDY FOR NON-CONFORMING PRODUCTS DURING THE WARRANTY PERIOD IS LIMITED TO REPAIR, REPLACEMENT OF OR REFUND FOR THE NON-CONFORMING PRODUCT(S) AT SELLER'S SOLE OPTION. THERE IS NO OBLIGATION TO REPAIR, REPLACE OR REFUND FOR PRODUCTS AS THE RESULT OF (I) ACCIDENT, DISASTER OR EVENT OF FORCE MAJEURE, (II) MISUSE, FAULT OR NEGLIGENCE OF OR BY BUYER, (III) USE OF THE PRODUCTS IN A MANNER FOR WHICH THEY WERE NOT DESIGNED, OR (IV) IMPROPER STORAGE AND HANDLING OF THE PRODUCTS. Unless otherwise expressly stated on the Product or in the documentation accompanying the Product, the Product is intended for research only and is not to be used for any other purpose, including without limitation, unauthorized commercial uses, in vitro diagnostic uses, ex vivo or in vivo therapeutic uses, or any type of consumption by or application to human or animals.

Human / Not Cited	13-6890 was used in blocking assay to determine the mechanism by which ceruloplasmin stimulates cellular iron uptake.<br><br>The Journal of biological chemistry (Jan 1999; 274: 1116)<br>**"Ceruloplasmin ferroxidase activity stimulates cellular iron uptake by a trivalent cation-specific transport mechanism."**<br>Author(s):Attieh ZK,Mukhopadhyay CK,Seshadri V,Tripoulas NA,Fox PL<br>PubMed Article URL:http://dx.doi.org/null
Mouse / Not Cited	13-6890 was used in western blot to elucidate major histocompatibility complex class I trafficking.<br><br>Traffic (Copenhagen, Denmark) (Feb 2001; 2: 124)<br>**"Antigen loading of MHC class I molecules in the endocytic tract."**<br>Author(s):Kleijmeer MJ,Escola JM,UytdeHaag FG,Jakobson E,Griffith JM,Osterhaus AD,Stoorvogel W,Melief CJ,Rabouille C, Geuze HJ<br>PubMed Article URL:http://dx.doi.org/null
Human / Not Cited	13-6890 was used in immunoprecipitation and western blot to elucidate the role of Rab15 in endocytosis.<br><br>The Journal of biological chemistry (Sep 2000; 275: 26754)<br>**"Rab15 differentially regulates early endocytic trafficking."**<br>Author(s):Zuk PA,Elferink LA<br>PubMed Article URL:http://dx.doi.org/10.1074/jbc.M000344200
Mouse / 1:50	13-6890 was used in immunocytochemistry to report that KIF2beta is a motor protein involved in peripheral translocation of lysosomes.<br><br>The EMBO journal (Oct 1998; 17: 5855)<br>**"KIF2beta, a new kinesin superfamily protein in non-neuronal cells, is associated with lysosomes and may be implicated in their centrifugal translocation."**<br>Author(s):Santama N,Krijnse-Locker J,Griffiths G,Noda Y,Hirokawa N,Dotti CG<br>PubMed Article URL:http://dx.doi.org/10.1093/emboj/17.20.5855
Yeast / 1:100	13-6890 was used in flow cytometry to perform high-throughput antibody screening using detergent-solubilized and biotinylated whole-cell lysates as the antigen source<br><br>Protein engineering, design and selection : PEDS (Jul 2010; 23: 567)<br>**"Antibody library screens using detergent-solubilized mammalian cell lysates as antigen sources."**<br>Author(s):Cho YK,Shusta EV<br>PubMed Article URL:http://dx.doi.org/10.1093/protein/gzq029
Human / Not Cited	13-6890 was used in western blot to test if Rab15 interfere with Rab5 function directly or indirectly.<br><br>The Journal of biological chemistry (Sep 2002; 277: 32722)<br>**"Mammalian suppressor of Sec4 modulates the inhibitory effect of Rab15 during early endocytosis."**<br>Author(s):Strick DJ,Francescutti DM,Zhao Y,Elferink LA<br>PubMed Article URL:http://dx.doi.org/10.1074/jbc.M205101200
Mouse / 1:500	13-6890 was used in western blot to report that the treatment of lung epithelial cells with BLM resulted in elevated intracellular Ca(2+) levels and stimulates PKC-1.<br><br>The international journal of biochemistry and cell biology (Mar 2012; 44: 514)<br>**"Activation of P2X7R and downstream effects in bleomycin treated lung epithelial cells."**<br>Author(s):Bläsche R,Ebeling G,Perike S,Weinhold K,Kasper M,Barth K<br>PubMed Article URL:http://dx.doi.org/10.1016/j.biocel.2011.12.003
Human / Not Cited	13-6890 was used in western blot to examine the roles of Rab3B and Rab13 in regulating the cell-surface transport of apical p75 neurotrophin receptor, basolateral low-density lipoprotein receptor, and Claudin-1.<br><br>Biochemical and biophysical research communications (Aug 2003; 308: 270)<br>**"Distinct roles of Rab3B and Rab13 in the polarized transport of apical, basolateral, and tight junctional membrane proteins to the plasma membrane."**<br>Author(s):Yamamoto Y,Nishimura N,Morimoto S,Kitamura H,Manabe S,Kanayama HO,Kagawa S,Sasaki T<br>PubMed Article URL:http://dx.doi.org/null
Human / Not Cited	13-6890 was used in western blot to report that the cytoplasmic domain of transferrin receptor interacts with GABARAP.<br><br>FEBS letters (May 2002; 518: 101)<br>**"Association of human transferrin receptor with GABARAP."**<br>Author(s):Green F,O'Hare T,Blackwell A,Enns CA<br>PubMed Article URL:http://dx.doi.org/null

For Research Use Only. Not for use in diagnostic procedures. Not for resale without express authorization.

Products are warranted to operate or perform substantially in conformance with published Product specifications in effect at the time of sale, as set forth in the Production documentation, specifications and/or accompanying package inserts ("Documentation"). No claim of suitability for use in applications regulated by FDA is made. The warranty provided herein is valid only when used by properly trained individuals. Unless otherwise stated in the Documentation, this warranty is limited to one year from date of shipment when the Product is subjected to normal, proper and intended usage. This warranty does not extend to anyone other than the Buyer. Any model or sample furnished to Buyer is merely illustrative of the general type and quality of goods and does not represent that any Product will conform to such model or sample.

NO OTHER WARRANTIES, EXPRESS OR IMPLED, ARE GRANTED INCLUDING WITHOUT LIMITATION, IMPLIED WARRANTIES OF MERCHANTABILITY, FITNESS FOR ANY PARTICULAR PURPOSE, OR NON INFRINGEMENT. BUYER'S EXCLUSIVE REMEDY FOR NON-CONFORMING PRODUCTS DURING THE WARRANTY PERIOD IS LIMITED TO REPAIR, REPLACEMENT OF OR REFUND FOR THE NON-CONFORMING PRODUCT(S) AT SELLER'S SOLE OPTION. THERE IS NO OBLIGATION TO REPAIR, REPLACE OR REFUND FOR PRODUCTS AS THE RESULT OF (I) ACCIDENT, DISASTER OR EVENT OF FORCE MAJEURE, (II) MISUSE, FAULT OR NEGLIGENCE OF OR BY BUYER, (III) USE OF THE PRODUCTS IN A MANNER FOR WHICH THEY WERE NOT DESIGNED, OR (IV) IMPROPER STORAGE AND HANDLING OF THE PRODUCTS. Unless otherwise expressly stated on the Product or in the documentation accompanying the Product, the Product is intended for research only and is not to be used for any other purpose, including without limitation, unauthorized commercial uses, in vitro diagnostic uses, ex vivo or in vivo therapeutic uses, or any type of consumption by or application to human or animals.

	13-6890 was used in immunocytochemistry to study the polymorphism of CD72 associated with susceptibility to human systemic lupus erythematosus.
Mouse / Not Cited	BMC immunology (Dec 2012; 13: null)<br>**"Human CD72 splicing isoform responsible for resistance to systemic lupus erythematosus regulates serum immunoglobulin level and is localized in endoplasmic reticulum."**<br>Author(s):Hitomi Y,Adachi T,Tsuchiya N,Honda Z,Tokunaga K,Tsubata T<br>PubMed Article URL:http://dx.doi.org/10.1186/1471-2172-13-72
	13-6890 was used in western blot to study the subcellular localization of human P4-ATPases and their interactions with CDC50 proteins.
Human / Not Cited	The Journal of biological chemistry (Nov 2011; 286: 38159)<br>**"ATP9B, a P4-ATPase (a putative aminophospholipid translocase), localizes to the trans-Golgi network in a CDC50 protein-independent manner."**<br>Author(s):Takatsu H,Baba K,Shima T,Umino H,Kato U,Umeda M,Nakayama K,Shin HW<br>PubMed Article URL:http://dx.doi.org/10.1074/jbc.M111.281006
	13-6890 was used in western blot to investigate factors that regulate ErbB2-ErbB3 heterodimerization.
Human / Not Cited	Carcinogenesis (Sep 2013; 34: 2031)<br>**"Pertuzumab counteracts the inhibitory effect of ErbB2 on degradation of ErbB3."**<br>Author(s):Sak MM,Szymanska M,Bertelsen V,Hasmann M,Madshus IH,Stang E<br>PubMed Article URL:http://dx.doi.org/10.1093/carcin/bgt173
	13-6890 was used in immunohistochemistry - paraffin section to study liver tumor promotion mechanisms of copper- and iron-overloading
Rat / 1:200	Chemico-biological interactions (May 2010; 185: 189)<br>**"Tumor promotion by copper-overloading and its enhancement by excess iron accumulation involving oxidative stress responses in the early stage of a rat two-stage hepatocarcinogenesis model."**<br>Author(s):Mizukami S,Ichimura R,Kemmochi S,Wang L,Taniai E,Mitsumori K,Shibutani M<br>PubMed Article URL:http://dx.doi.org/10.1016/j.cbi.2010.03.023
	13-6890 was used in western blot to explore the role of zinc in vascular dysfunction.
Human / Not Cited	PloS one (Aug 2012; 7: null)<br>**"Angiotensin II requires zinc and downregulation of the zinc transporters ZnT3 and ZnT10 to induce senescence of vascular smooth muscle cells."**<br>Author(s):Patrushev N,Seidel-Rogol B,Salazar G<br>PubMed Article URL:http://dx.doi.org/10.1371/journal.pone.0033211
	13-6890 was used in immunocytochemistry to identify and characterize aczonin.
Mouse / Not Cited	The Journal of cell biology (Oct 1999; 147: 151)<br>**"Aczonin, a 550-kD putative scaffolding protein of presynaptic active zones, shares homology regions with Rim and Bassoon and binds profilin."**<br>Author(s):Wang X,Kibschull M,Laue MM,Lichte B,Petrasch-Parwez E,Kilimann MW<br>PubMed Article URL:http://dx.doi.org/null
	13-6890 was used in immunocytochemistry to investigate the transfer of endocytosed EGFR from endosomes to lysosomes.
Mouse / Not Cited	The Journal of biological chemistry (Mar 2004; 279: 11562)<br>**"Sorting of ligand-activated epidermal growth factor receptor to lysosomes requires its actin-binding domain."**<br>Author(s):Stoorvogel W,Kerstens S,Fritzsche I,den Hartigh JC,Oud R,van der Heyden MA,Voortman J,van Bergen en Henegouwen PM<br>PubMed Article URL:http://dx.doi.org/10.1074/jbc.M308449200
	13-6890 was used in immunohistochemistry - paraffin section to examine Transferrin Receptor 1 expression in IUGR and control placentas
Human / Not Cited	Placenta (Jan 2011; 32: 44)<br>**"Transferrin receptor gene and protein expression and localization in human IUGR and normal term placentas."**<br>Author(s):Mandò C,Tabano S,Colapietro P,Pileri P,Colleoni F,Avagliano L,Doi P,Bulfamante G,Miozzo M,Cetin I<br>PubMed Article URL:http://dx.doi.org/10.1016/j.placenta.2010.10.009

For Research Use Only. Not for use in diagnostic procedures. Not for resale without express authorization.

Products are warranted to operate or perform substantially in conformance with published Product specifications in effect at the time of sale, as set forth in the Production documentation, specifications and/or accompanying package inserts ("Documentation"). No claim of suitability for use in applications regulated by FDA is made. The warranty provided herein is valid only when used by properly trained individuals. Unless otherwise stated in the Documentation, this warranty is limited to one year from date of shipment when the Product is subjected to normal, proper and intended usage. This warranty does not extend to anyone other than the Buyer. Any model or sample furnished to Buyer is merely illustrative of the general type and quality of goods and does not represent that any Product will conform to such model or sample.

NO OTHER WARRANTIES, EXPRESS OR IMPLED, ARE GRANTED INCLUDING WITHOUT LIMITATION, IMPLIED WARRANTIES OF MERCHANTABILITY, FITNESS FOR ANY PARTICULAR PURPOSE, OR NON INFRINGEMENT. BUYER'S EXCLUSIVE REMEDY FOR NON-CONFORMING PRODUCTS DURING THE WARRANTY PERIOD IS LIMITED TO REPAIR, REPLACEMENT OF OR REFUND FOR THE NON-CONFORMING PRODUCT(S) AT SELLER'S SOLE OPTION. THERE IS NO OBLIGATION TO REPAIR, REPLACE OR REFUND FOR PRODUCTS AS THE RESULT OF (I) ACCIDENT, DISASTER OR EVENT OF FORCE MAJEURE, (II) MISUSE, FAULT OR NEGLIGENCE OF OR BY BUYER, (III) USE OF THE PRODUCTS IN A MANNER FOR WHICH THEY WERE NOT DESIGNED, OR (IV) IMPROPER STORAGE AND HANDLING OF THE PRODUCTS. Unless otherwise expressly stated on the Product or in the documentation accompanying the Product, the Product is intended for research only and is not to be used for any other purpose, including without limitation, unauthorized commercial uses, in vitro diagnostic uses, ex vivo or in vivo therapeutic uses, or any type of consumption by or application to human or animals.

	13-6890 was used in western blot to examine the in vivo the regulation of DGK alpha.
Human / Not Cited	Journal of immunology (Baltimore, Md. : 1950) (Mar 2003; 170: 2877)<br>**"T cell activation in vivo targets diacylglycerol kinase alpha to the membrane: a novel mechanism for Ras attenuation."**<br>Author(s):Sanjuán MA,Pradet-Balade B,Jones DR,Martínez-A C,Stone JC,Garcia-Sanz JA,Mérida I<br>PubMed Article URL:http://dx.doi.org/null
	13-6890 was used in western blot to determine the role of Fer protein-tyrosine kinase in non-small cell lung cancer tumor progression.
Human / 1:1000	Molecular cancer research : MCR (Aug 2013; 11: 952)<br>**"Fer protein-tyrosine kinase promotes lung adenocarcinoma cell invasion and tumor metastasis."**<br>Author(s):Ahn J,Truesdell P,Meens J,Kadish C,Yang X,Boag AH,Craig AW<br>PubMed Article URL:http://dx.doi.org/10.1158/1541-7786.MCR-13-0003-T
	13-6890 was used in western blot to examine the role of deubiquitylating enzymes in cholesterol metabolism
Human / 1:1000	The Journal of biological chemistry (Feb 2016; 291: 4813)<br>**"Deubiquitylase Inhibition Reveals Liver X Receptor-independent Transcriptional Regulation of the E3 Ubiquitin Ligase IDOL and Lipoprotein Uptake."**<br>Author(s):Nelson JK,Cook EC,Loregger A,Hoeksema MA,Scheij S,Kovacevic I,Hordijk PL,Ovaa H,Zelcer N<br>PubMed Article URL:http://dx.doi.org/10.1074/jbc.M115.698688
	13-6890 was used in western blot to elucidate the regulation of the endocytic pathways by ARF6.
Human / Not Cited	The Journal of biological chemistry (Jul 1999; 274: 20040)<br>**"Distribution of ARF6 between membrane and cytosol is regulated by its GTPase cycle."**<br>Author(s):Gaschet J,Hsu VW<br>PubMed Article URL:http://dx.doi.org/null
	13-6890 was used in immunoprecipitation to determine which domains of class I heavy chains bind to US11.
Human / Not Cited	European journal of immunology (Jun 2003; 33: 1707)<br>**"Amino acid composition of alpha1/alpha2 domains and cytoplasmic tail of MHC class I molecules determine their susceptibility to human cytomegalovirus US11-mediated down-regulation."**<br>Author(s):Barel MT,Pizzato N,van Leeuwen D,Bouteiller PL,Wiertz EJ,Lenfant F<br>PubMed Article URL:http://dx.doi.org/10.1002/eji.200323912
	13-6890 was used in western blot to investigate mechanisms that regulate of ADAM10.
Mouse / Not Cited	Cellular and molecular life sciences : CMLS (Sep 2012; 69: 2919)<br>**"Tetraspanin15 regulates cellular trafficking and activity of the ectodomain sheddase ADAM10."**<br>Author(s):Prox J,Willenbrock M,Weber S,Lehmann T,Schmidt-Arras D,Schwanbeck R,Saftig P,Schwake M<br>PubMed Article URL:http://dx.doi.org/10.1007/s00018-012-0960-2
	13-6890 was used in western blot to elucidate the caveolin-1-mediated senescence in bleomycin treatment of A549 cells
Human / 1:500	The international journal of biochemistry and cell biology (Jan 2011; 43: 98)<br>**"Bleomycin treatment of A549 human lung cancer cells results in association of MGr1-Ag and caveolin-1 in lipid rafts."**<br>Author(s):Linge A,Meleady P,Henry M,Clynes M,Kasper M,Barth K<br>PubMed Article URL:http://dx.doi.org/10.1016/j.biocel.2010.10.001
	13-6890 was used in flow cytometry to determine a role of red blood cells in T-cell proliferation.
Human / Not Cited	Blood (May 2001; 97: 3152)<br>**"Red blood cells inhibit activation-induced cell death and oxidative stress in human peripheral blood T lymphocytes."**<br>Author(s):Fonseca AM,Porto G,Uchida K,Arosa FA<br>PubMed Article URL:http://dx.doi.org/null
	13-6890 was used in immunocytochemistry to show that Rab14 binds in a GTP-dependent manner to RUFY1/Rabip4
Human / Not Cited	Molecular biology of the cell (Aug 2010; 21: 2746)<br>**"Functional cross-talk between Rab14 and Rab4 through a dual effector, RUFY1/Rabip4."**<br>Author(s):Yamamoto H,Koga H,Katoh Y,Takahashi S,Nakayama K,Shin HW<br>PubMed Article URL:http://dx.doi.org/10.1091/mbc.e10-01-0074

For Research Use Only. Not for use in diagnostic procedures. Not for resale without express authorization.

Products are warranted to operate or perform substantially in conformance with published Product specifications in effect at the time of sale, as set forth in the Production documentation, specifications and/or accompanying package inserts ("Documentation"). No claim of suitability for use in applications regulated by FDA is made. The warranty provided herein is valid only when used by properly trained individuals. Unless otherwise stated in the Documentation, this warranty is limited to one year from date of shipment when the Product is subjected to normal, proper and intended usage. This warranty does not extend to anyone other than the Buyer. Any model or sample furnished to Buyer is merely illustrative of the general type and quality of goods and does not represent that any Product will conform to such model or sample.

NO OTHER WARRANTIES, EXPRESS OR IMPLED, ARE GRANTED INCLUDING WITHOUT LIMITATION, IMPLIED WARRANTIES OF MERCHANTABILITY, FITNESS FOR ANY PARTICULAR PURPOSE, OR NON INFRINGEMENT. BUYER'S EXCLUSIVE REMEDY FOR NON-CONFORMING PRODUCTS DURING THE WARRANTY PERIOD IS LIMITED TO REPAIR, REPLACEMENT OF OR REFUND FOR THE NON-CONFORMING PRODUCT(S) AT SELLER'S SOLE OPTION. THERE IS NO OBLIGATION TO REPAIR, REPLACE OR REFUND FOR PRODUCTS AS THE RESULT OF (I) ACCIDENT, DISASTER OR EVENT OF FORCE MAJEURE, (II) MISUSE, FAULT OR NEGLIGENCE OF OR BY BUYER, (III) USE OF THE PRODUCTS IN A MANNER FOR WHICH THEY WERE NOT DESIGNED, OR (IV) IMPROPER STORAGE AND HANDLING OF THE PRODUCTS. Unless otherwise expressly stated on the Product or in the documentation accompanying the Product, the Product is intended for research only and is not to be used for any other purpose, including without limitation, unauthorized commercial uses, in vitro diagnostic uses, ex vivo or in vivo therapeutic uses, or any type of consumption by or application to human or animals.

Non-human primate / Not Cited	13-6890 was used in immunocytochemistry to identify the intramolecular region involved in the specific localization of NHE6 and NHE7<br><br>The Journal of membrane biology (Apr 2010; 234: 149)<br>**"A membrane-proximal region in the C-terminal tail of NHE7 is required for its distribution in the trans-Golgi network, distinct from NHE6 localization at endosomes."**<br>Author(s):Fukura N,Ohgaki R,Matsushita M,Nakamura N,Mitsui K,Kanazawa H<br>PubMed Article URL:http://dx.doi.org/10.1007/s00232-010-9242-9
Human / Not Cited	13-6890 was used in western blot to elucidate the mechanism of Saponinum album-induced cytotoxicity.<br><br>Molecular pharmaceutics (Dec 2011; 8: 2262)<br>**"The endocytic uptake pathways of targeted toxins are influenced by synergistically acting Gypsophila saponins."**<br>Author(s):Bachran D,Schneider S,Bachran C,Weng A,Melzig MF,Fuchs H<br>PubMed Article URL:http://dx.doi.org/10.1021/mp200130j
Mouse / Not Cited	13-6890 was used in western blot to study the downregulation and intracellular trafficking of the cocaine- and amphetamine-sensitive dopamine transporter.<br><br>The Journal of neuroscience : the official journal of the Society for Neuroscience (Sep 1999; 19: 7699)<br>**"Membrane trafficking regulates the activity of the human dopamine transporter."**<br>Author(s):Melikian HE,Buckley KM<br>PubMed Article URL:http://dx.doi.org/null
Human / 1:1000	13-6890 was used in western blot to identify 4-hydroxynonenal as a hemozoin-generated inhibitory molecule and describe molecular targets of 4-hydroxynonena in erythroid progenitors<br><br>Blood (Nov 2010; 116: 4328)<br>**"Inhibition of erythropoiesis in malaria anemia: role of hemozoin and hemozoin-generated 4-hydroxynonenal."**<br>Author(s):Skorokhod OA,Caione L,Marrocco T,Migliardi G,Barrera V,Arese P,Piacibello W,Schwarzer E<br>PubMed Article URL:http://dx.doi.org/10.1182/blood-2010-03-272781
Human / 1:1000	13-6890 was used in immunohistochemistry - paraffin section to define the immunohistochemical profile of CD71<br><br>American journal of clinical pathology (Sep 2010; 134: 429)<br>**"CD71 (transferrin receptor): an effective marker for erythroid precursors in bone marrow biopsy specimens."**<br>Author(s):Marsee DK,Pinkus GS,Yu H<br>PubMed Article URL:http://dx.doi.org/10.1309/AJCPCRK3MOAOJ6AT
Mouse / Not Cited	13-6890 was used in immunocytochemistry to identify cellular changes induced byTOR1A mutations.<br><br>Human molecular genetics (May 2000; 9: 1403)<br>**"Mutant torsinA, responsible for early-onset torsion dystonia, forms membrane inclusions in cultured neural cells."**<br>Author(s):Hewett J,Gonzalez-Agosti C,Slater D,Ziefer P,Li S,Bergeron D,Jacoby DJ,Ozelius LJ,Ramesh V,Breakefield XO<br>PubMed Article URL:http://dx.doi.org/null
Human / 1:200	13-6890 was used in western blot to show that that Gal3 functionally integrates carbohydrate specificity on cargo proteins.<br><br>Nature cell biology (Jun 2014; 16: 595)<br>**"Galectin-3 drives glycosphingolipid-dependent biogenesis of clathrin-independent carriers."**<br>Author(s):Lakshminarayan R,Wunder C,Becken U,Howes MT,Benzing C,Arumugam S,Sales S,Ariotti N,Chambon V,Lamaze C,Loew D,Shevchenko A,Gaus K,Parton RG,Johannes L<br>PubMed Article URL:http://dx.doi.org/10.1038/ncb2970
Mouse / Not Cited	13-6890 was used in immunoprecipitation to elucidate the interactions among US2/HLA-A2/beta(2)-microglobulin.<br><br>Journal of immunology (Baltimore, Md. : 1950) (Dec 2003; 171: 6757)<br>**"Human cytomegalovirus-encoded US2 differentially affects surface expression of MHC class I locus products and targets membrane-bound, but not soluble HLA-G1 for degradation."**<br>Author(s):Barel MT,Ressing M,Pizzato N,van Leeuwen D,Le Bouteiller P,Lenfant F,Wiertz EJ<br>PubMed Article URL:http://dx.doi.org/null
Human / Not Cited	13-6890 was used in western blot to study the turnover of intact cell-surface slit diaphragm components.<br><br>Journal of biochemistry (Aug 2014; 156: 115)<br>**"aPKC maintains the integrity of the glomerular slit diaphragm through trafficking of nephrin to the cell surface."**<br>Author(s):Satoh D,Hirose T,Harita Y,Daimon C,Harada T,Kurihara H,Yamashita A,Ohno S<br>PubMed Article URL:http://dx.doi.org/10.1093/jb/mvu022

For Research Use Only. Not for use in diagnostic procedures. Not for resale without express authorization.

Products are warranted to operate or perform substantially in conformance with published Product specifications in effect at the time of sale, as set forth in the Production documentation, specifications and/or accompanying package inserts ("Documentation"). No claim of suitability for use in applications regulated by FDA is made. The warranty provided herein is valid only when used by properly trained individuals. Unless otherwise stated in the Documentation, this warranty is limited to one year from date of shipment when the Product is subjected to normal, proper and intended usage. This warranty does not extend to anyone other than the Buyer. Any model or sample furnished to Buyer is merely illustrative of the general type and quality of goods and does not represent that any Product will conform to such model or sample.

NO OTHER WARRANTIES, EXPRESS OR IMPLED, ARE GRANTED INCLUDING WITHOUT LIMITATION, IMPLIED WARRANTIES OF MERCHANTABILITY, FITNESS FOR ANY PARTICULAR PURPOSE, OR NON INFRINGEMENT. BUYER'S EXCLUSIVE REMEDY FOR NON-CONFORMING PRODUCTS DURING THE WARRANTY PERIOD IS LIMITED TO REPAIR, REPLACEMENT OF OR REFUND FOR THE NON-CONFORMING PRODUCT(S) AT SELLER'S SOLE OPTION. THERE IS NO OBLIGATION TO REPAIR, REPLACE OR REFUND FOR PRODUCTS AS THE RESULT OF (I) ACCIDENT, DISASTER OR EVENT OF FORCE MAJEURE, (II) MISUSE, FAULT OR NEGLIGENCE OF OR BY BUYER, (III) USE OF THE PRODUCTS IN A MANNER FOR WHICH THEY WERE NOT DESIGNED, OR (IV) IMPROPER STORAGE AND HANDLING OF THE PRODUCTS. Unless otherwise expressly stated on the Product or in the documentation accompanying the Product, the Product is intended for research only and is not to be used for any other purpose, including without limitation, unauthorized commercial uses, in vitro diagnostic uses, ex vivo or in vivo therapeutic uses, or any type of consumption by or application to human or animals.

Human / 1:1500	13-6890 was used in western blot to discuss genes that are involved in Long QT syndrome.<br><br>Circulation. Cardiovascular genetics (Jun 2013; 6: 279)<br>**"Exome sequencing and systems biology converge to identify novel mutations in the L-type calcium channel, CACNA1C, linked to autosomal dominant long QT syndrome."**<br>Author(s):Boczek NJ,Best JM,Tester DJ,Giudicessi JR,Middha S,Evans JM,Kamp TJ,Ackerman MJ<br>PubMed Article URL:http://dx.doi.org/10.1161/CIRCGENETICS.113.000138
Human / Not Cited	13-6890 was used in western blot to demonstrate a critical role for the GPCR proteolysis site in autoproteolysis that mediates receptor signaling and cell activation.<br><br>Molecular and cellular biology (Apr 2012; 32: 1408)<br>**"Activation of myeloid cell-specific adhesion class G protein-coupled receptor EMR2 via ligation-induced translocation and interaction of receptor subunits in lipid raft microdomains."**<br>Author(s):Huang YS,Chiang NY,Hu CH,Hsiao CC,Cheng KF,Tsai WP,Yona S,Stacey M,Gordon S,Chang GW,Lin HH<br>PubMed Article URL:http://dx.doi.org/10.1128/MCB.06557-11
Human / Not Cited	13-6890 was used in western blot to determine the localization of potassium channels in placental tissue.<br><br>The Journal of membrane biology (Mar 2012; 245: 141)<br>**"Differential expression of potassium channels in placentas from normal and pathological pregnancies: targeting of the K(ir) 2.1 channel to lipid rafts."**<br>Author(s):Riquelme G,de Gregorio N,Vallejos C,Berrios M,Morales B<br>PubMed Article URL:http://dx.doi.org/10.1007/s00232-012-9422-x
Horse / 1:25	13-6890 was used in immunohistochemistry - paraffin section to test if equine TfR expression correlates with a regenerative response in anemic horses<br><br>Veterinary clinical pathology (Dec 2010; 39: 424)<br>**"Cloning and tissue expression of the equine transferrin receptor."**<br>Author(s):Webb TL,Burnett RC,Avery AC,Olver CS<br>PubMed Article URL:http://dx.doi.org/10.1111/j.1939-165X.2010.00265.x
Human / 1 µg	13-6890 was used in immunoprecipitation to report that RAP binds to and inhibits Abeta.<br><br>Journal of neurochemistry (Mar 2010; 112: 1199)<br>**"Inhibition of Abeta aggregation and neurotoxicity by the 39-kDa receptor-associated protein."**<br>Author(s):Kerr ML,Gasperini R,Gibbs ME,Hou X,Shepherd CE,Strickland DK,Foa L,Lawen A,Small DH<br>PubMed Article URL:http://dx.doi.org/10.1111/j.1471-4159.2009.06540.x
Human / Not Cited	13-6890 was used in immunohistochemistry to investigate the expression of the transferrin receptor 1, proliferating cell nuclear antigen, p27, and calbindin in feline panleukopenia virus negative kittens.<br><br>Veterinary journal (London, England : 1997) (Jun 2013; 196: 388)<br>**"Expression of transferrin receptor 1, proliferating cell nuclear antigen, p27(Kip1) and calbindin in the fetal and neonatal feline cerebellar cortex."**<br>Author(s):Poncelet L,Springinsfeld M,Ando K,Héraud C,Kabova A,Brion JP<br>PubMed Article URL:http://dx.doi.org/10.1016/j.tvjl.2012.10.017
Human / Not Cited	13-6890 was used in western blot to study the effect of retromer mutations on endosome-to-TGN transport.<br><br>Current biology : CB (Jul 2014; 24: 1670)<br>**"Retromer binding to FAM21 and the WASH complex is perturbed by the Parkinson disease-linked VPS35(D620N) mutation."**<br>Author(s):McGough IJ,Steinberg F,Jia D,Barbuti PA,McMillan KJ,Heesom KJ,Whone AL,Caldwell MA,Billadeau DD,Rosen MK,Cullen PJ<br>PubMed Article URL:http://dx.doi.org/10.1016/j.cub.2014.06.024
Human / Not Cited	13-6890 was used in western blot to investigate the role of HFE in iron regulation.<br><br>The Journal of biological chemistry (Aug 1998; 273: 22068)<br>**"Co-trafficking of HFE, a nonclassical major histocompatibility complex class I protein, with the transferrin receptor implies a role in intracellular iron regulation."**<br>Author(s):Gross CN,Irrinki A,Feder JN,Enns CA<br>PubMed Article URL:http://dx.doi.org/null

5 Flow Cytometry References

Species / Dilution	Summary

For Research Use Only. Not for use in diagnostic procedures. Not for resale without express authorization.

Products are warranted to operate or perform substantially in conformance with published Product specifications in effect at the time of sale, as set forth in the Production documentation, specifications and/or accompanying package inserts ("Documentation"). No claim of suitability for use in applications regulated by FDA is made. The warranty provided herein is valid only when used by properly trained individuals. Unless otherwise stated in the Documentation, this warranty is limited to one year from date of shipment when the Product is subjected to normal, proper and intended usage. This warranty does not extend to anyone other than the Buyer. Any model or sample furnished to Buyer is merely illustrative of the general type and quality of goods and does not represent that any Product will conform to such model or sample.

NO OTHER WARRANTIES, EXPRESS OR IMPLED, ARE GRANTED INCLUDING WITHOUT LIMITATION, IMPLIED WARRANTIES OF MERCHANTABILITY, FITNESS FOR ANY PARTICULAR PURPOSE, OR NON INFRINGEMENT. BUYER'S EXCLUSIVE REMEDY FOR NON-CONFORMING PRODUCTS DURING THE WARRANTY PERIOD IS LIMITED TO REPAIR, REPLACEMENT OF OR REFUND FOR THE NON-CONFORMING PRODUCT(S) AT SELLER'S SOLE OPTION. THERE IS NO OBLIGATION TO REPAIR, REPLACE OR REFUND FOR PRODUCTS AS THE RESULT OF (I) ACCIDENT, DISASTER OR EVENT OF FORCE MAJEURE, (II) MISUSE, FAULT OR NEGLIGENCE OF OR BY BUYER, (III) USE OF THE PRODUCTS IN A MANNER FOR WHICH THEY WERE NOT DESIGNED, OR (IV) IMPROPER STORAGE AND HANDLING OF THE PRODUCTS. Unless otherwise expressly stated on the Product or in the documentation accompanying the Product, the Product is intended for research only and is not to be used for any other purpose, including without limitation, unauthorized commercial uses, in vitro diagnostic uses, ex vivo or in vivo therapeutic uses, or any type of consumption by or application to human or animals.

Not Applicable / Not Cited	13-6890 was used in flow cytometry and western blot to report that the human herpesvirus-7 U21 gene product interferes with natural killer cell recognition<br><br>PLoS pathogens (Nov 2011; 7: null)<br>**"The human herpesvirus-7 (HHV-7) U21 immunoevasin subverts NK-mediated cytoxicity through modulation of MICA and MICB."**<br>Author(s):Schneider CL,Hudson AW<br>PubMed Article URL:http://dx.doi.org/10.1371/journal.ppat.1002362
Hamster / Not Cited	13-6890 was used in flow cytometry to study Nef inhibitors as a means to control HIV infection.<br><br>PloS one (Oct 2011; 6: null)<br>**"Molecular design, functional characterization and structural basis of a protein inhibitor against the HIV-1 pathogenicity factor Nef."**<br>Author(s):Breuer S,Schievink SI,Schulte A,Blankenfeldt W,Fackler OT,Geyer M<br>PubMed Article URL:http://dx.doi.org/10.1371/journal.pone.0020033
Not Applicable / Not Cited	13-6890 was used in flow cytometry to identify the residues responsible for the resistance or sensitivity of MHC class I molecules to US2- and US11-mediated down-regulation<br><br>International immunology (Jan 2006; 18: 173)<br>**"Subtle sequence variation among MHC class I locus products greatly influences sensitivity to HCMV US2- and US11-mediated degradation."**<br>Author(s):Barel MT,Pizzato N,Le Bouteiller P,Wiertz EJ,Lenfant F<br>PubMed Article URL:http://dx.doi.org/10.1093/intimm/dxh362
Not Applicable / Not Cited	13-6890 was used in flow cytometry, immunocytochemistry, and western blot to elucidate the physiological function of TSAP6<br><br>Cell death and differentiation (Nov 2008; 15: 1723)<br>**"Exosome secretion, including the DNA damage-induced p53-dependent secretory pathway, is severely compromised in TSAP6/Steap3-null mice."**<br>Author(s):Lespagnol A,Duflaut D,Beekman C,Blanc L,Fiucci G,Marine JC,Vidal M,Amson R,Telerman A<br>PubMed Article URL:http://dx.doi.org/10.1038/cdd.2008.104
Not Applicable / Not Cited	13-6890 was used in flow cytometry and western blot to propose that transferrin receptor expression is involved in the regulation of ultraviolet light-resistance<br><br>Journal of radiation research (Dec 2005; 46: 443)<br>**"Enhanced expression of transferrin receptor confers UV-resistance in human and monkey cells."**<br>Author(s):Chen Z,Nomura J,Suzuki T,Suzuki N<br>PubMed Article URL:http://dx.doi.org/null

1 Immunoprecipitation References

Species / Dilution	Summary
Not Applicable / Not Cited	13-6890 was used in immunoprecipitation and western blot to study the regulation and function of soluble beta2microglobulin-HFE monochain and transferrin receptor<br><br>Journal of cellular biochemistry (Apr 2004; 91: 1130)<br>**"Transferrin [corrected] receptor association and endosomal localization of soluble HFE are not sufficient for regulation of cellular iron homeostasis."**<br>Author(s):Laham N,Rotem-Yehudar R,Shechter C,Coligan JE,Ehrlich R<br>PubMed Article URL:http://dx.doi.org/10.1002/jcb.20015

For Research Use Only. Not for use in diagnostic procedures. Not for resale without express authorization.

Products are warranted to operate or perform substantially in conformance with published Product specifications in effect at the time of sale, as set forth in the Production documentation, specifications and/or accompanying package inserts ("Documentation"). No claim of suitability for use in applications regulated by FDA is made. The warranty provided herein is valid only when used by properly trained individuals. Unless otherwise stated in the Documentation, this warranty is limited to one year from date of shipment when the Product is subjected to normal, proper and intended usage. This warranty does not extend to anyone other than the Buyer. Any model or sample furnished to Buyer is merely illustrative of the general type and quality of goods and does not represent that any Product will conform to such model or sample.

NO OTHER WARRANTIES, EXPRESS OR IMPLED, ARE GRANTED INCLUDING WITHOUT LIMITATION, IMPLIED WARRANTIES OF MERCHANTABILITY, FITNESS FOR ANY PARTICULAR PURPOSE, OR NON INFRINGEMENT. BUYER'S EXCLUSIVE REMEDY FOR NON-CONFORMING PRODUCTS DURING THE WARRANTY PERIOD IS LIMITED TO REPAIR, REPLACEMENT OF OR REFUND FOR THE NON-CONFORMING PRODUCT(S) AT SELLER'S SOLE OPTION. THERE IS NO OBLIGATION TO REPAIR, REPLACE OR REFUND FOR PRODUCTS AS THE RESULT OF (I) ACCIDENT, DISASTER OR EVENT OF FORCE MAJEURE, (II) MISUSE, FAULT OR NEGLIGENCE OF OR BY BUYER, (III) USE OF THE PRODUCTS IN A MANNER FOR WHICH THEY WERE NOT DESIGNED, OR (IV) IMPROPER STORAGE AND HANDLING OF THE PRODUCTS. Unless otherwise expressly stated on the Product or in the documentation accompanying the Product, the Product is intended for research only and is not to be used for any other purpose, including without limitation, unauthorized commercial uses, in vitro diagnostic uses, ex vivo or in vivo therapeutic uses, or any type of consumption by or application to human or animals.

Thermo Fisher Scientific
3747 N. Meridian Road
Rockford, IL 61105 USA

thermofisher.com/contactus

ThermoFisher SCIENTIFIC
