# Supplementary material for: Evaluation of TFR-1 Expression in Feline Mammary Cancer and In Vitro Antitumor Efficacy Study of Doxorubicin-Loaded H-Ferritin Nanocages
Source: Cancers (Basel). 2021 Mar 12;13(6):1248. doi: 10.3390/cancers13061248 (PMC8000254; doi:10.3390/cancers13061248)
Supplement: Supplementary file 1 [file cancers-13-01248-s001.zip › Figure S5.pdf]

SANTA CRUZ BIOTECHNOLOGY, INC.

β-Actin (C4): sc-47778

SANTA CRUZ BIOTECHNOLOGY
The Power to Question

BACKGROUND

All eukaryotic cells express Actin, which often constitutes as much as 50% of total cellular protein. Actin filaments can form both stable and labile structures and are crucial components of microvilli and the contractile apparatus of muscle cells. While lower eukaryotes, such as yeast, have only one Actin gene, higher eukaryotes have several isoforms encoded by a family of genes. At least six types of Actin are present in mammalian tissues and fall into three classes. α-Actin expression is limited to various types of muscle, whereas β-Actin and γ-Actin are the principle constituents of filaments in other tissues. Members of the small GTPase family regulate the organization of the Actin cytoskeleton. Rho controls the assembly of Actin stress fibers and focal adhesion. Rac regulates Actin filament accumulation at the plasma membrane. Cdc42 stimulates formation of filopodia.

REFERENCES

1. Lessard, J.L. 1988. Two monoclonal antibodies to actin: one muscle selective and one generally reactive. Cell Motil. Cytoskeleton 10: 349-362.
2. Doolittle, R.F. 1995. The origins and evolution of eukaryotic proteins. Philos. Trans. R. Soc. Lond., B, Biol. Sci. 349: 235-240.
3. Maccioni, R.B., et al. 1995. Role of microtubule-associated proteins in the control of microtubule assembly. Physiol. Rev. 75: 835-864.

CHROMOSOMAL LOCATION

Genetic locus: ACTB (human) mapping to 7p22.1; Actb (mouse) mapping to 5 G2.

SOURCE

β-Actin (C4) is a mouse monoclonal antibody raised against gizzard Actin of chicken origin.

PRODUCT

Each vial contains 200 µg IgG_1 kappa light chain in 1.0 ml of PBS with < 0.1% sodium azide and 0.1% gelatin. Also available as TransCruz reagent for Gel Supershift and ChIP applications, sc-47778 X, 200 µg/0.1 ml.

β-Actin (C4) is available conjugated to agarose (sc-47778 AC), 500 µg/0.25 ml agarose in 1 ml, for IP; to HRP (sc-47778 HRP), 200 µg/ml, for WB, IHC(P) and ELISA; to either phycoerythrin (sc-47778 PE), fluorescein (sc-47778 FITC), Alexa Fluor® 488 (sc-47778 AF488), Alexa Fluor® 546 (sc-47778 AF546), Alexa Fluor® 594 (sc-47778 AF594) or Alexa Fluor® 647 (sc-47778 AF647), 200 µg/ml, for WB (RGB), IF, IHC(P) and FCM; and to either Alexa Fluor® 680 (sc-47778 AF680) or Alexa Fluor® 790 (sc-47778 AF790), 200 µg/ml, for Near-Infrared (NIR) WB, IF and FCM.

In addition, β-Actin (C4) is available conjugated to biotin (sc-47778 B), 200 µg/ml, for WB, IHC(P) and ELISA.

Alexa Fluor® is a trademark of Molecular Probes, Inc., Oregon, USA

STORAGE

Store at 4° C, **DO NOT FREEZE**. Stable for one year from the date of shipment. Non-hazardous. No MSDS required.

APPLICATIONS

β-Actin (C4) is recommended for detection of β-Actin of mouse, rat, human, avian, bovine, canine, porcine, rabbit, *Dictyostelium discoideum* and *Physarum polycephalum* origin by Western Blotting (starting dilution 1:200, dilution range 1:100-1:1000), immunoprecipitation [1-2 µg per 100-500 µg of total protein (1 ml of cell lysate)], immunofluorescence (starting dilution 1:50, dilution range 1:50-1:500), immunohistochemistry (including paraffin-embedded sections) (starting dilution 1:50, dilution range 1:50-1:500) and solid phase ELISA (starting dilution 1:30, dilution range 1:30-1:3000); may cross-react with all six known isoforms of Actin in higher vertebrates (including cytoplasmic β- and γ-Actin isoforms, skeletal, cardiac, and vascular α-Actin isoforms, and enteric γ-Actin isoform).

Suitable for use as control antibody for β-Actin siRNA (h): sc-108069, β-Actin siRNA (m): sc-108070, β-Actin siRNA (r): sc-156106, β-Actin shRNA Plasmid (h): sc-108069-SH, β-Actin shRNA Plasmid (m): sc-108070-SH, β-Actin shRNA Plasmid (r): sc-156106-SH, β-Actin shRNA (h) Lentiviral Particles: sc-108069-V, β-Actin shRNA (m) Lentiviral Particles: sc-108070-V and β-Actin shRNA (r) Lentiviral Particles: sc-156106-V.

β-Actin (C4) X TransCruz antibody is recommended for Gel Supershift and ChIP applications.

Molecular Weight of β-Actin/C-terminal region of β-Actin: 43/15 kDa.

DATA

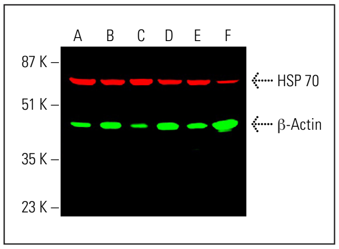

Simultaneous infrared western blot analysis of β-Actin expression, detected with β-Actin (C4): sc-47778 and m-IgGκ BP-CFL 680: sc-516180 and HSP 70/HSC 70 expression, detected with HSP 70/HSC 70 (W27): sc-24 and m-IgGλ BP-CFL 790: sc-516195 in HeLa (**A**), DU 145 (**B**), MCF7 (**C**), MDA-MB-231 (**D**), HCT-116 (**E**) and HUV-EC-C (**F**) whole cell lysates.

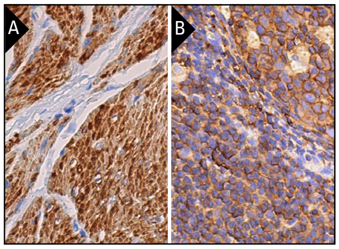

β-Actin (C4): sc-47778. Immunoperoxidase detection of β-Actin in formalin fixed, paraffin-embedded human smooth muscle tissue, showing cytoplasmic staining of smooth muscle cells. Detection reagent used: m-IgGκ BP-HRP: sc-516102 (**A**). Immunoperoxidase detection of β-Actin in formalin fixed, paraffin-embedded human tonsil tissue, showing cytoplasmic and membrane staining of cells in germinal center and cells in non-germinal center. Detection reagent used: m-IgGκ BP-HRP: sc-516102 (**B**).

SELECT PRODUCT CITATIONS

1. Teicher, M.H. and Barber, N.I. 1990. COSIFIT: an interactive program for simultaneous multioscillator cosinor analysis of time-series data. Comput. Biomed. Res. 23: 283-295.
2. Zou, Z., et al. 2021. MAP4K4 induces early blood-brain barrier damage in a murine subarachnoid hemorrhage model. Neural. Regen. Res. 16: 325-332.

RESEARCH USE

For research use only, not for use in diagnostic procedures.
