# Supplementary figures and images for: Evaluation of TFR-1 Expression in Feline Mammary Cancer and In Vitro Antitumor Efficacy Study of Doxorubicin-Loaded H-Ferritin Nanocages
Source: Cancers (Basel). 2021 Mar 12;13(6):1248. doi: 10.3390/cancers13061248 (PMC8000254; doi:10.3390/cancers13061248)

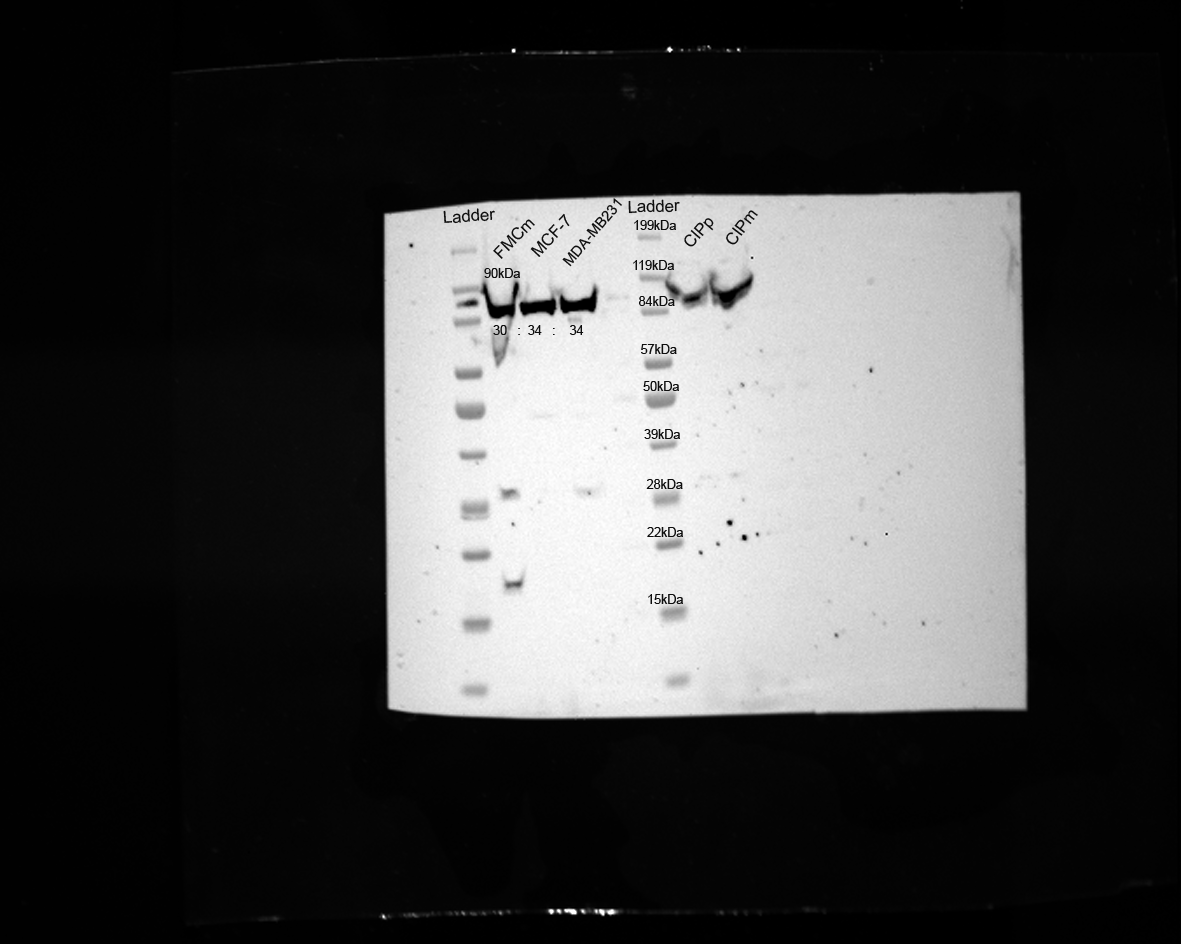

Supplement: Supplementary file 1 [file cancers-13-01248-s001.zip › Figure S2.tif]

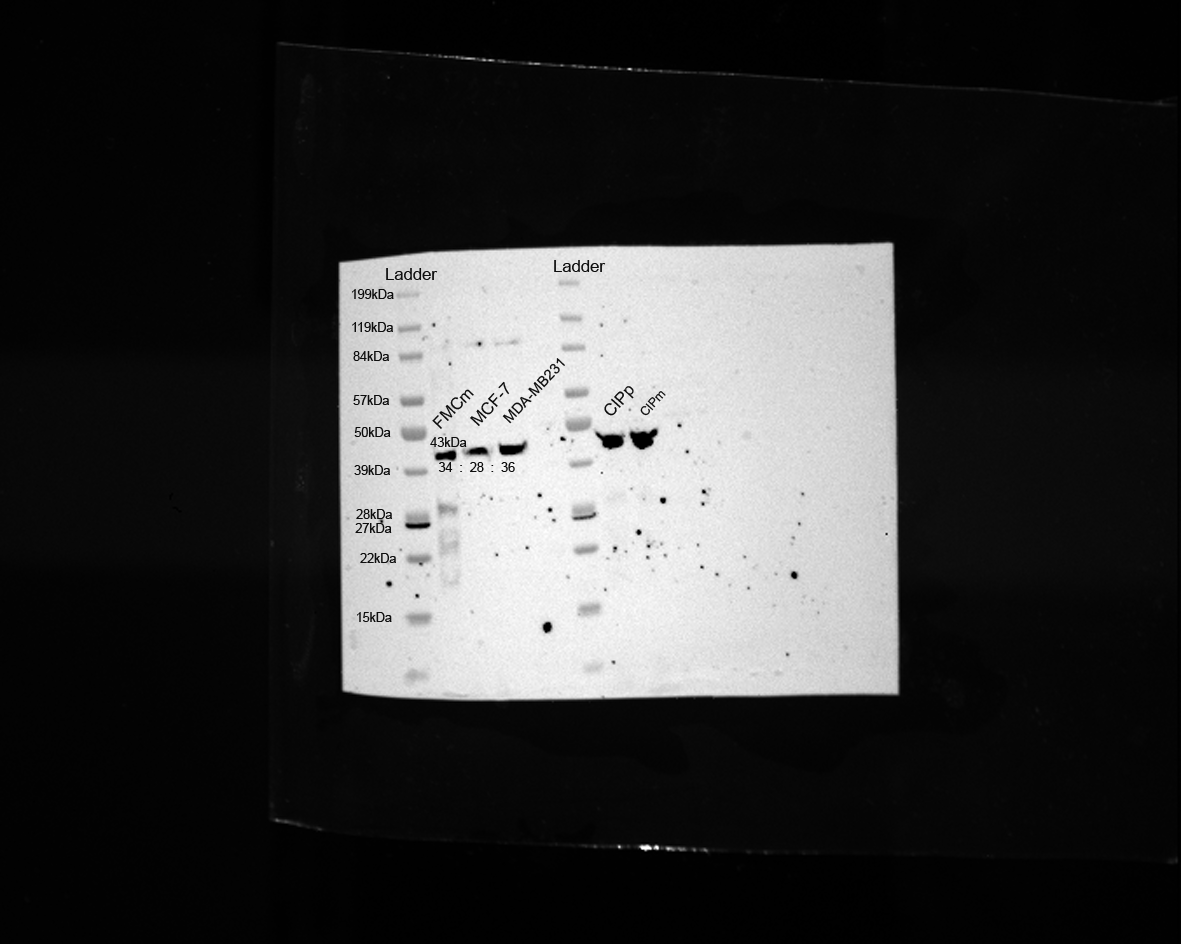

Supplement: Supplementary file 1 [file cancers-13-01248-s001.zip › Figure S3.tif]
